# Direct and Low‐Temperature Regeneration of Degraded LiFePO₄ Cathodes at Ambient Conditions Using Green and Sustainable Deep Eutectic Solvent

**DOI:** 10.1002/advs.202504683

**Published:** 2025-05-20

**Authors:** Yixin Lin, Tiansheng Wang, Chaochao Gao, Xiaoxuan Zhang, Wen Yu, Mi Wang, Chao Yang, Jiaheng Zhang

**Affiliations:** ^1^ School of Science Harbin Institute of Technology (Shenzhen) Shenzhen 518055 China; ^2^ School of Materials Science and Engineering Harbin Institute of Technology (Shenzhen) Shenzhen 518055 China; ^3^ Research Centre of Printed Flexible Electronics Harbin Institute of Technology (Shenzhen) Shenzhen 518055 China; ^4^ Sauvage Laboratory for Smart Materials School of Materials Science and Engineering Harbin Institute of Technology (Shenzhen) Shenzhen 518055 China; ^5^ Shenzhen Shinehigh Innovation technology Ltd. Taoyuan Street, Nanshan District Shenzhen 518055 China; ^6^ Department of Chemistry University of Idaho Moscow ID 83844‐2343 USA

**Keywords:** deep eutectic solvent, direct regeneration, Li–Fe antisite defects, lithium iron phosphate batteries, reducing environment

## Abstract

The definite lifespan of lithium iron phosphate (LiFePO_4_, LFP) batteries necessitates the advancement of cost‐effective, nature‐friendly, and productive recycling techniques for spent LFP batteries. In this study, ethylene glycol (C_2_H_6_O_2_), a sustainable and economical small organic molecule, is employed as a multifunctional hydrogen‐bonding donor, along with lithium chloride (LiCl), a readily accessible Li source and hydrogen‐bonding acceptor. Together, they form a novel Li‐salt deep eutectic solvent (DES) through hydrogen bonding interactions. This DES directly repairs and rejuvenates the spent cathode material (S‐LFP) at 80 °C. The Li‐salt DES not only replenishes the depleted Li in S‐LFP and reduces the adverse effects of Li–Fe antisite defects but also establishes a reducing environment that facilitates the reversion of degraded Fe(III) species in S‐LFP back to their original Fe(II) state. Consequently, the regenerated LFP exhibits remarkable electrochemical behavior, delivering an initial capacity of 155.6 mAh g^−1^ at 0.1 C and retaining 93% of its initial capacity after 300 cycles at 1 C. This approach can be scaled up to treat large quantities of LFP cathode material recovered from fully retired batteries, presenting a practical pathway toward large‐scale recycling of spent LFP batteries in the future.

## Graphical abstract

Computational predictions identify ethylene glycol and lithium chloride as optimal components for a green deep eutectic solvent (DES). And further investigations determine the optimal reaction conditions and process parameters for DES treatment of spent lithium iron phosphate (S‐LFP). This solvent enables direct regeneration of S‐LFP at 80 °C, effectively mitigating lithium deficiency and Fe(II) oxidation while repairing Li–Fe antisite defects.

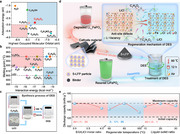

## 1 Introduction

In recent years, advancements in Li‐ion batteries have successfully achieved the dual objectives of enhanced safety and increased energy density, significantly broadening their potential applications.^[^ ^1^, ^2^, ^3^ ^]^ Li iron phosphate (LFP) batteries, which utilize LiFePO₄ as the cathode material, are extensively employed in electric transportation and aerospace sectors owing to their high structural and thermal stability, prolonged cycle life, and affordability.^[^ ^4^, ^5^ ^]^ Data reveal that the electric transportation industry has propelled the consistent rise in Li‐ion battery installations, highlighting the critical role of LFP and ternary Li batteries in the Li‐ion market.^[^ ^6^, ^7^, ^8^ ^]^ However, with the typical lifespan of traditional Li‐ion batteries ranging only from five to eight years, their extensive deployment is anticipated to cause a substantial increase in the accumulated volume of retired batteries in the future.^[^ ^9^ ^]^ Consequently, managing and recycling spent Li‐ion batteries have emerged as crucial issues, underlining the necessity for effective disposal and recovery techniques as a primary research focus.

The conventional recycling of spent lithium‐ion batteries primarily employs hydrometallurgical and pyrometallurgical approaches to recover critical metals, such as lithium, from cathode materials. These methods, while effective in metal extraction, often face challenges related to environmental impact and process efficiency, highlighting the need for more sustainable recycling solutions. However, these processes are energy‐intensive and complex and typically require acidic or alkaline leaching agents to extract only trace amounts of Li.^[^ ^10^ ^]^ These methods yield low economic returns and can lead to secondary pollution due to chemical reagents.^[^ ^11^, ^12^, ^13^ ^]^ As a result, such techniques are suboptimal for the mainstream recycling of degraded Li‐ion batteries, driving interest to direct regeneration methods.

Direct regeneration offers a potent approach for restoring the electrochemical performance of spent batteries; however, a thorough comprehension of the failure mechanisms of Li‐ion batteries is crucial for optimizing their effectiveness. Typically, the capacity loss in cathode materials over long‐term cycling primarily arises from the depletion of active Li, irreversible phase changes, and damage to the crystal structure.^[^ ^14^, ^15^ ^]^ In the case of LFP materials, degradation is caused by the loss of active Li or antisite defects between Li and Fe.^[^ ^16^ ^]^ Recent studies on direct solid‐phase regeneration methods have concentrated on LFP batteries. These studies involve adding Li sources such as Li carbonate and reducing agents such as ammonium hydrogen phosphate and sucrose to degraded S‐LFP powders. These mixtures are then calcined at high temperatures to yield regenerated LFP powder.^[^ ^17^, ^18^ ^]^ However, accurately controlling the Li‐to‐iron atomic ratio is challenging, complicating the restoration of S‐LFP powders with varying degradation levels. Researchers have resorted to hydrothermal methods for direct regeneration to overcome these challenges. These methods enable soluble Li salts to effectively contact S‐LFP powders in solution, aided by reducing agents, followed by brief annealing to efficiently regenerate the material. For instance, Xu et al.^[^ ^19^ ^]^ restored spent LFP by immersing it in a solution containing Li hydroxide and citric acid, followed by hydrothermal lithiation and brief annealing at 600 °C. Similarly, Tang et al.^[^ ^9^ ^]^ effectively restored S‐LFP using cost‐effective, naturally derived ‐threonine and CH₃COOLi at a low temperature of 180 °C. The hydrothermal approach offers several benefits, including the avoidance of precise Li stoichiometry calculations, reduced energy consumption, and preservation of the original LFP structure, establishing it as a promising area for research Li^[^ ^20^, ^21^ ^]^ into the direct regeneration of spent Li‐ion batteries.

Building on the foundation of hydrothermal methods, we developed a Li‐containing deep eutectic solvent (DES) to directly regenerate S‐LFPs at low temperatures. This novel green solvent offers significant advantages such as straightforward synthesis, cost‐effectiveness, low volatility, strong solvating capabilities, and environmental sustainability.^[^ ^22^, ^23^, ^24^, ^25^ ^]^ Moreover, it effectively regenerates S‐LFP at low temperatures, representing an optimized and advanced approach to hydrothermal regeneration. Previously, Fei et al. synthesized a ternary DES using betaine as the hydrogen bond acceptor (HBA) and Li ethylene glycol urea as the hydrogen bond donor (HBD) to restore spent LiCoO₂ cathode materials through a 10 h stirring process at 120 °C.^[^ ^26^ ^]^ Similarly, Yu et al. developed a ternary Li DES by bonding inorganic salts LiOH and LiNO₃ with Li salicylate (LSA) to regenerate ternary LiNiMnCo_2_ cathode materials, demonstrating the feasibility of direct DES regeneration of spent batteries.^[^ ^27^ ^]^ However, the direct regeneration of S‐LFPs using DES remains largely unexplored.^[^ ^28^, ^29^, ^30^ ^]^ Given the significant market demand for LFP batteries, designing a Li‐containing DES for the direct regeneration of LFP could yield substantial economic benefits and reduce the natural effect of battery squander.

This study introduces a novel green Li‐containing DES created by combining ethylene glycol, a small organic molecule serving as an HBD, with Li chloride, which acts as the HBA. This DES facilitates the direct regeneration of S‐LFP powder at low temperatures, achieving efficient recovery of the spent LFP cathode material. Ethylene glycol, as the HBD, and Li chloride, as the HBA, engage in hydrogen bonding that lowers the eutectic point of the DES to below 100 °C. This reduction enables selective diffusion of Li⁺ into the vacancies within the S‐LFP lattice at these low temperatures. Additionally, the reducing conditions established by the DES restore Fe^3+^ phase to its original Fe^2+^ phase state and alleviate Li–Fe antisite defects, thereby improving Li⁺ transport. As a result, the DES‐regenerated R‐LFP delivers an initial discharge capacity of 155.6 mAh g^−1^ at 0.1 C and retains 93% of this capacity after 300 cycles at 1 C. This strategy has also proven effective for S‐LFP samples with varying degrees of degradation and composition, yielding regenerated R‐LFP whose electrochemical performance is comparable to or surpasses commercial standards. Moreover, the recyclability and reusability of the DES have been verified, maximizing its economic advantages and offering a sustainable, low‐energy, and scalable solution for recycling spent LFP batteries.

## 2 Results and Discussion

### 2.1 Design and Synthesis of DES for Direct Regeneration of S‐LFP

To achieve the regeneration and restoration of spent lithium iron phosphate (S‐LFP), an ideal DES must exhibit specific functional properties, including precise lithium replenishment at lithium‐deficient sites, reduction of the degraded Fe(III) phase, and correction of Li–Fe antisite defects. Consequently, the selection of hydrogen bond donors (HBDs) and hydrogen bond acceptors (HBAs) for DES synthesis must adhere to strict criteria. A suitable HBD should allow Li^+^ to maintain sufficient mobility within the system, facilitating its directed diffusion into S‐LFP for lithium supplementation. Additionally, previous studies suggest that the repair process requires a reducing atmosphere, which not only promotes the spontaneous diffusion of Li^+^ into S‐LFP but also facilitates the reduction of Fe(III) to Fe(II) and the correction of Li–Fe antisite defects.^[^ ^22^, ^31^ ^]^ Based on these criteria, we calculated the adsorption energy of Li^+^ with common HBDs to evaluate its diffusion tendency within the system. Furthermore, we computed the highest occupied molecular orbital (HOMO) energy levels of different HBDs to quantify their electron‐donating capability, thereby assessing their reducing potential (computational models are shown in Figure S4, Supporting Information).^[^ ^32^, ^33^ ^]^

**Figure 1:**
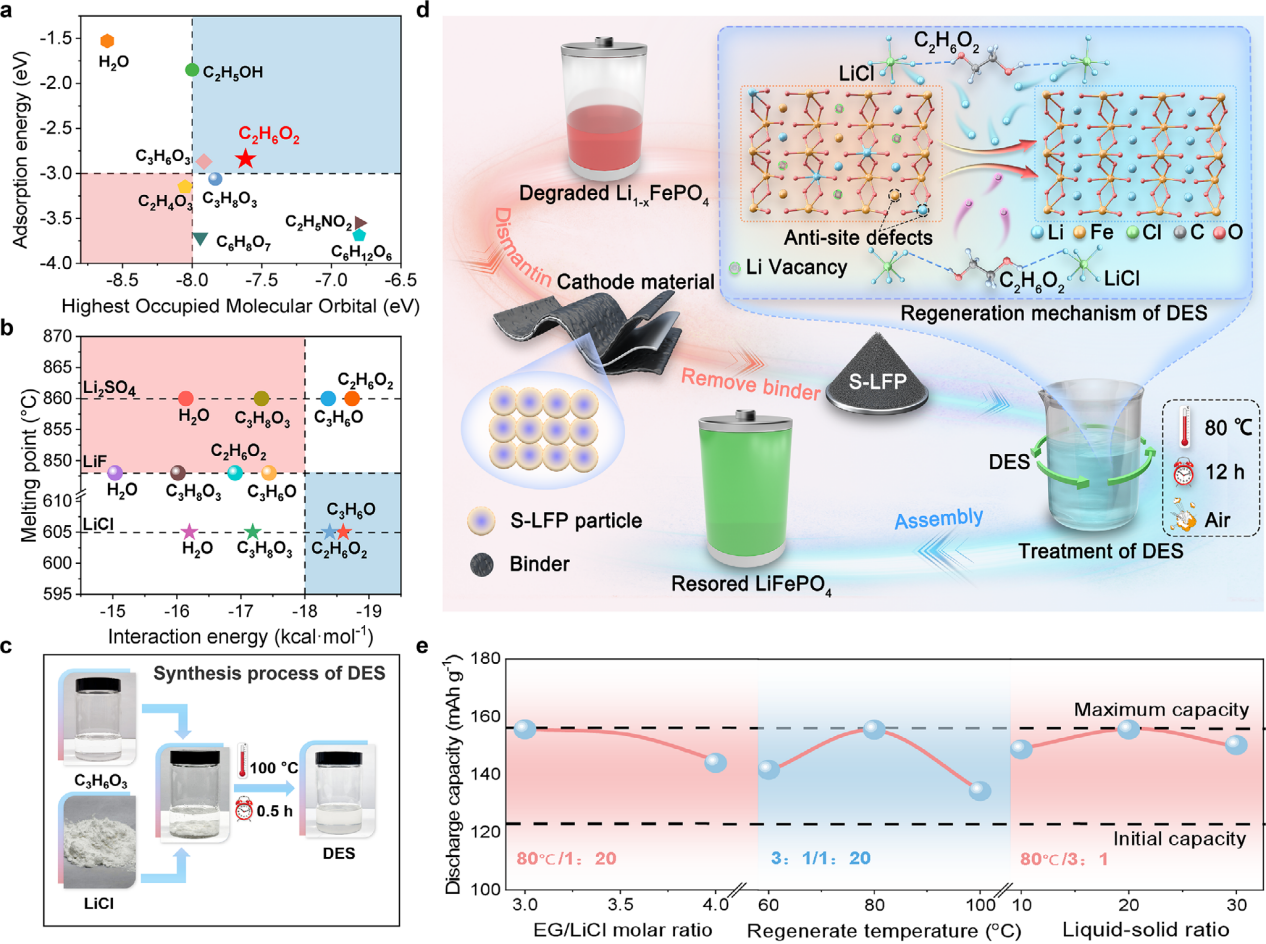
Direct regeneration strategy of S‐LFP using DES and its design concept. a) Screening of HBD components for DES synthesis. b) Screening of HBA components for DES synthesis. c) The synthesis process of DES. d) Mechanism of direct regeneration of S‐LFP by DES. e) Battery performance under various regeneration conditions.

As illustrated in **Figure** **1**a, computational predictions indicate that H_2_O, ethanol (EtOH), ethylene glycol (EG), and glycerol (GLY) are potential HBD capable of facilitating S‐LFP restoration. To validate their practical effectiveness, we conducted a preliminary assessment of these four candidates. H_2_O exhibits excellent Li^+^ transport properties; however, its lack of reducing capability may compromise the effectiveness of S‐LFP restoration. EtOH provides both Li^+^ transport and moderate reducing ability; however, its low boiling point results in significant evaporation during water bath synthesis, affecting its practical applicability. GLY has high viscosity and strong Li^+^ adsorption, which limits Li^+^ diffusion in the system, leading to reduced efficacy in practical applications. EG demonstrates both effective Li^+^ transport and a suitable reducing atmosphere, without exhibiting major limitations under experimental conditions. Therefore, EG was identified as the most promising HBD.

Despite the identification of an optimal HBD, synthesizing a DES that directly incorporates lithium salts remains challenging due to the high melting points and poor solubility of common lithium salts in most solvents. To address this issue, we sought to identify an HBA that is well‐matched with the selected HBD, which could potentially overcome these limitations and facilitate the synthesis of a stable lithium‐containing DES. Accordingly, we calculated the intermolecular interactions between common lithium salts (LiCl, LiF, and Li_2_SO_4_) and the four candidate HBDs (H_2_O, EtOH, EG, and GLY) to evaluate their molecular stability in the DES system (computational models are shown in Figure S3, Supporting Information). Additionally, by considering the intrinsic melting points of lithium salts, we assessed whether the selected HBD–HBA combinations could form stable DESs at lower temperatures.

As depicted in Figure 1b, among the tested lithium salts, LiCl exhibited the strongest interaction energy with the predicted HBD, making it the most promising candidate for forming a stable lithium salt complex. Furthermore, LiCl has a relatively low melting point, which reduces the thermal energy required for DES synthesis, thereby enhancing the feasibility of preparing lithium‐containing DES. Moreover, given that all selected HBD are polar molecules, LiCl exhibits significantly higher solubility in polar solvents compared to LiF and Li_2_SO_4_, making it the most viable option for DES synthesis.^[^ ^34^ ^]^

Building on the computational predictions and practical feasibility assessments of HBDs and HBAs, we proceeded with the synthesis of multiple DES candidates. As demonstrated in Figure S5 of the Supporting Information, experimental results revealed that when Li_2_SO_4_ and LiF were used as HBAs, the resulting mixtures remained in a solid powder state under identical reaction conditions. This was attributed to their poor solubility in polar solvents and intrinsically high melting points, which hindered DES formation. When ethanol was used as the HBD, only lithium salt powder remained in the reaction vessel after a certain period, indicating that ethanol had evaporated during the water bath heating process. Only the LiCl–H_2_O, LiCl–EG, and LiCl–GLY systems formed clear and transparent solutions, with no solid‐phase precipitation after cooling to room temperature. To confirm DES formation, we conducted Fourier‐transform infrared (FT‐IR) spectroscopy on these three solutions. As shown in Figure S6 of the Supporting Information, all three systems exhibited significant red shifts in the IR spectra, indicating successful DES formation. However, comparative analysis revealed that the LiCl–GLY system exhibited significantly higher viscosity (Figure S7, Supporting Information). Given that excessive viscosity could hinder Li^+^ diffusion during the S‐LFP restoration process, leading to reduced repair efficiency, and considering that the computational predictions had already indicated lower Li^+^ transport and reducing capability for LiCl–GLY compared to LiCl–EG, the LiCl–GLY system was deemed unsuitable as an ideal DES candidate.

To further confirm the optimal DES formulation among the remaining two candidates, we conducted preliminary S‐LFP restoration experiments using LiCl–H_2_O and LiCl–EG DES systems under identical repair conditions. As shown in Figure S8 of the Supporting Information, the R‐LFP battery restored using the LiCl–EG DES exhibited significantly superior capacity performance compared to that restored using LiCl–H_2_O. This discrepancy arises because, in the LiCl–H_2_O system, the HBD H_2_O lacks reducing ability, which prevents the spontaneous diffusion of Li^+^ into S‐LFP and fails to restore the degraded Fe(III) phase, ultimately leading to poor restoration efficiency. This result is consistent with our previous computational predictions regarding H_2_O's limitations. Based on these findings, LiCl–EG was identified as the optimal DES system, as it effectively facilitates Li^+^ transport and provides a suitable reducing atmosphere, thereby promoting the structural recovery and electrochemical performance enhancement of S‐LFP.^[^ ^35^ ^]^ These results offer a promising solvent system for the efficient regeneration of spent lithium iron phosphate batteries.

Based on this theoretical framework, we confirmed the selection of LiCl as the HBA and EG as the HBD in our DES system. By modifying the ratio of EG to LiCl and the synthesis temperature, we successfully lowered the eutectic point of the LiCl–EG DES system to below 100 °C, synthesizing a clear DES solution (Figure 1c; Figure S1, Supporting Information). To validate the DES, we first measured the melting point of the clarified solution using differential scanning calorimetry (DSC). The significant depression of the melting points of the two components used to synthesize the solution, as per the definition of a DES, indicates that LiCl and EG effectively formed a DES (Figure S9, Supporting Information). Further verification was provided by FT‐IR analysis (Figure S6a, Supporting Information) of the DES,^[^ ^31^ ^]^ which revealed intensified intramolecular and intermolecular hydrogen bonding between the two components, resulting in a notable shift in the vibration peak near 3400 cm^−1^. As the intramolecular hydrogen bonds transitioned to intermolecular hydrogen bonds, certain characteristic peaks broadened and strengthened while retaining the original peaks of the glycol solvent, confirming the formation of the LiCl–EG DES. Thermal stability and sustainability were also evaluated using thermogravimetric analysis (TG). The TG results indicated that, aside from minimal moisture evaporation from LiCl, heating the DES to 120 °C caused negligible weight loss after lowering the eutectic point to 80 °C (Figure S10, Supporting Information), demonstrating excellent thermal stability. The TG‐IR results shown in Figure S12 of the Supporting Information further confirmed the stability of the DES. These findings suggest that our optimized Li‐containing DES, with a eutectic point of 80 °C, is suitable for direct low‐temperature regeneration of degraded LFP cathode. The reduced eutectic point also enhances the diffusion rate of Li^+^ throughout the solvent system, as evidenced in Figure S9 of the Supporting Information.

This Li‐containing DES was specifically developed to facilitate the low‐temperature regeneration of spent LiFePO₄ (S‐LFP) batteries. A series of regeneration experiments were conducted under various conditions. As depicted in Figure 1e, the DES, formulated at a 3:1 molar ratio of glycol to LiCl and utilized alongside an 80 °C water bath and a 20:1 DES‐to‐S‐LFP mass ratio, yielded R‐LFP with a discharge capacity of up to 155.6 mAh g^−1^ at a 0.1 C rate. Under these parameters, the DES exhibited optimal regeneration performance for S‐LFP (see Figure S11, and Table S1, Supporting Information). The environmentally friendly attributes of this solvent also prompted an evaluation of its potential for side reactions. FT‐IR analysis of the DES, post‐regeneration of spent LFP at various temperatures (Figure S13, Supporting Information), revealed that, apart from minor peak shifts attributed to Li^+^ migration into the spent powder, the DES maintained most of the original spectral features, indicating minimal side reactions. The analysis suggested that the regenerated DES was essentially a Li‐depleted version of the original (Figure S16, Supporting Information). The recycled DES was reapplied in subsequent S‐LFP regeneration trials, demonstrating consistent regenerative effectiveness over three cycles (Figure S15, Supporting Information), thus confirming the recyclability of this DES and its potential for scalable, sustainable regeneration of spent LFP batteries.

To further investigate the influence of DES on the overall effectiveness of S‐LFP restoration and its role in the repair process, we conducted additional experiments using solvent systems with similar compositions to DES but without actual DES formation. These experiments were performed under identical molar ratios, temperatures, and other experimental conditions as described previously. As shown in Figure S17 of the Supporting Information, LiCl and EG were used to restore S‐LFP at varying reaction temperatures without forming DES. Since these two components function independently within the system, LiCl serves as the lithium source for replenishing lithium‐deficient sites in S‐LFP, while EG provides the reducing environment necessary for the restoration process. While this solvent system demonstrated a certain degree of repair capability, the electrochemical performance of the restored R‐LFP–LiCl/EG still exhibited a substantial gap compared to commercial‐grade LFP (C‐LFP). In addition, we selected LiF, a lithium source with physicochemical properties similar to LiCl, to examine its potential in S‐LFP restoration using EG as the solvent. However, due to the higher electronegativity of F^−^, the release of Li^+^ from LiF into the system and its subsequent migration into S‐LFP requires overcoming a significantly higher energy barrier. This indicates that, under the same low‐temperature and low‐pressure conditions, Li replenishment is less efficient, leading to inferior restoration performance that does not meet the standard of C‐LFP.

A comparative analysis of the capacity retention and rate performance of the restored R‐LFP samples from these four systems revealed that the LiCl–EG system, when forming DES, exhibited the best restoration effectiveness, even surpassing the performance of C‐LFP. This enhancement can be attributed to the synergistic effect arising from DES formation. At the onset of the restoration process, the reducing atmosphere provided by DES facilitates the release of Li^+^ from the solvent system under mild reaction conditions, enabling its spontaneous diffusion into S‐LFP. Simultaneously, this process promotes the reduction of the degraded Fe(III) phase back to Fe(II) and facilitates the correction of Li–Fe antisite defects.^[^ ^36^ ^]^ This synergistic effect significantly improves both the efficiency and effectiveness of S‐LFP restoration, even under identical solid‐to‐liquid ratios, reaction times, and temperatures.

These findings highlight the broader potential applications of DES synthesized from carefully selected hydrogen bond donors and acceptors for direct, low‐temperature, low‐pressure regeneration of spent S‐LFP, paving the way for more efficient and sustainable battery recycling strategies.

### 2.2 Molecular Dynamics Simulation of S‐LFP Regeneration Process

**Figure 2:**
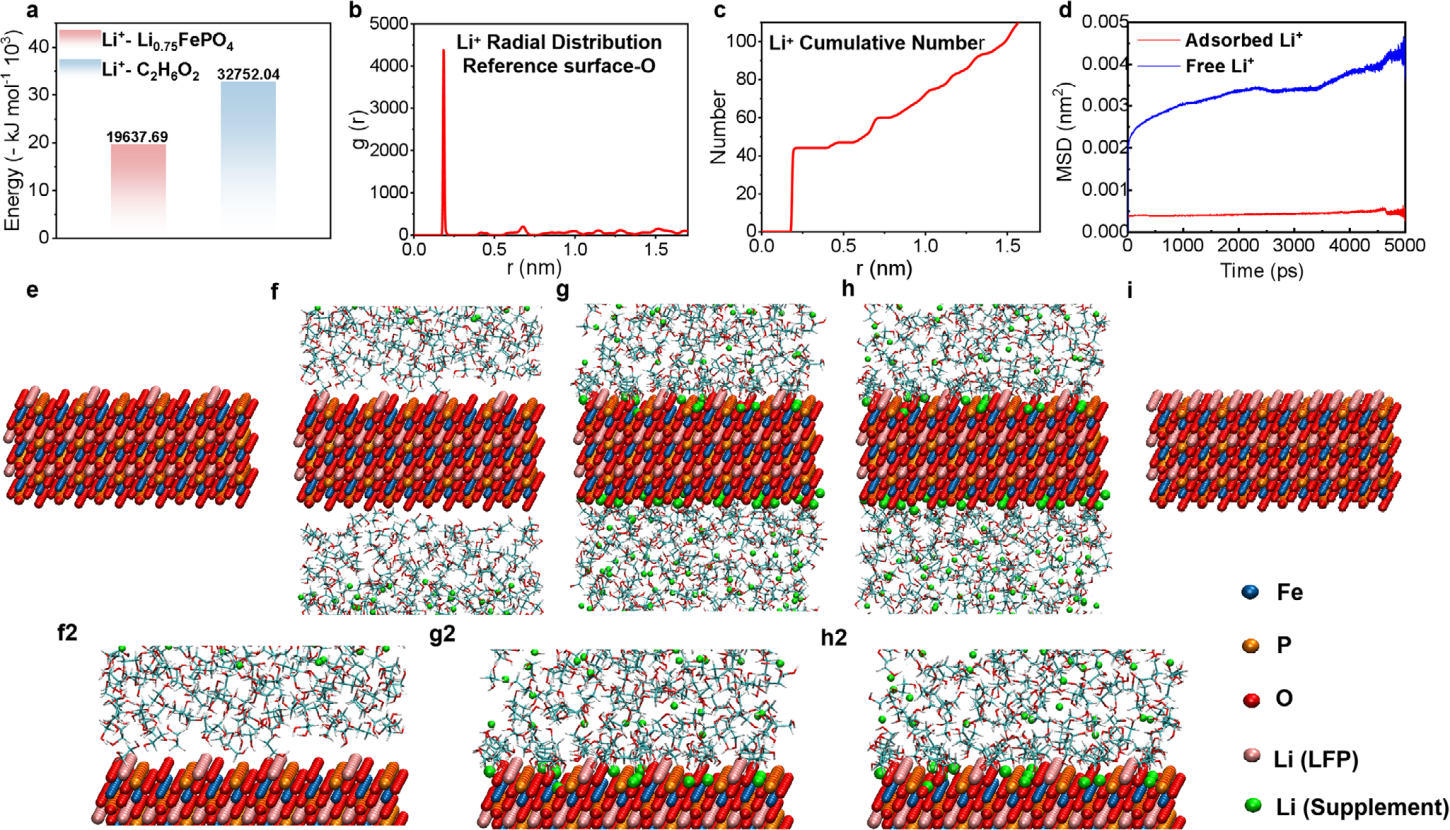
Molecular dynamics simulation analysis of the DES regeneration method for S‐LFP. a) Intermolecular energy of Li^+^ within the system. b) RDF of Li^+^ relative to oxygen atoms on the Li_0.75_FePO_4_ surface. c) Density distribution of Li^+^ ions. d) Mean square displacement curves for adsorbed and free Li^+^ on the Li_0.75_FePO_4_ material. e–i) Adsorption and regeneration process of Li_0.75_FePO_4_ illustrating the progressive incorporation of Li⁺ and structural repair.

Molecular dynamics simulations were applied to further analyze and predict the coupling interactions between the LiCl–C₂H₆O₂ DES system and S‐LFP (Li₀.₇₅FePO₄). Building on previous findings, these simulations explored the interactions at the interface.^[^ ^32^, ^34^, ^35^ ^]^ As illustrated in **Figure** **2**a, the intermolecular energy between Li^+^ and Li_0.75_FePO_4_ was −19637.69 kJ mol^−^¹, higher than that with ethylene glycol at −32752.04 kJ mol^−^¹. This suggests a preferential migration of Li⁺ ions from the DES, diffusing into Li‐deficient vacancies in Li_0.75_FePO_4_, while ethylene glycol remains stable (Figure S14, Supporting Information), aligning with initial predictions. The radial distribution function (RDF) was employed to analyze the proximity of oxygen atoms on the Li_0.75_FePO_4_ surface and Li^+^ ions in the solution. A pronounced peak at ≈0.2 nm confirms the availability of Li^+^ ions to the LiFePO₄ surface, aiding the repair process (Figure 2b).^[^ ^33^ ^]^ Furthermore, system analysis, as detailed in Figure 2c and Table S16 (Supporting Information), showed successful localization of over 40 Li^+^ ions on the Li_0.75_FePO_4_ surface, effectively filling vacancies and ensuring the stability of the adsorbed Li⁺ ions, substantiating their critical role in the process.

To evaluate this, we applied the following equation

(1) $$D=\frac{1}{6}\lim_{t\to \infty }\frac{d}{\mathrm{dt}}{\left|r\left(t\right)-r\left(0\right)\right|}^{2}$$

The self‐diffusion coefficients (*D*) of free and adsorbed Li^+^ ions were calculated to be 2.948 × 10⁻⁵ cm^2^ s^−1^ and 4.032 × 10⁻⁵ cm^2^ s^−1^, correspondingly (Figure 2c). This observation implies that free Li^+^ ions readily diffused into the Li_0.75_FePO_4_ structure, while the mobility of adsorbed Li^+^ was restricted. The consistent influx of external Li^+^, coupled with the stable presence of internal Li^+^, facilitates effective Li replenishment in S‐LFP. By scrutinizing the Li^+^ diffusion process, it was determined that the regeneration of S‐LFP material transpires as Li^+^ ions migrate from the solvent to the material's surface and subsequently integrate into the lattice, culminating in structural repair and regeneration, as depicted in Figure 4e–i.

### 2.3 Electrochemical Performance of Regenerated LFP Batteries

**Figure 3:**
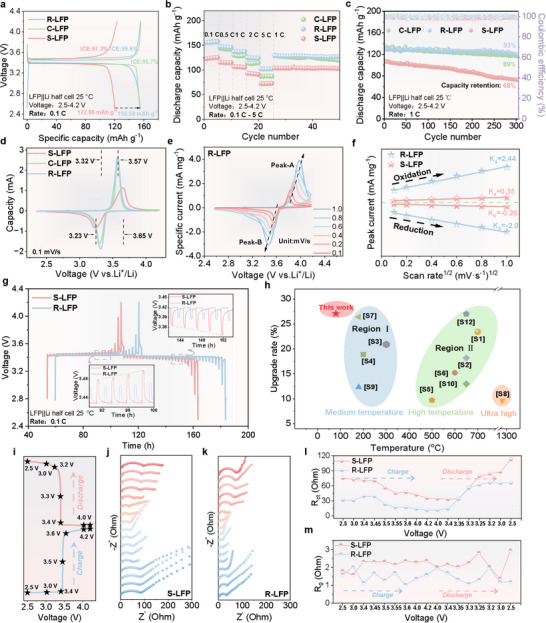
Electrochemical performance and kinetic characterization of LFP batteries. a) Initial charge–discharge profiles of S‐LFP, R‐LFP, and C‐LFP batteries. b) Rate performance of S‐LFP, R‐LFP, and C‐LFP batteries. c) Cycling stability of S‐LFP, R‐LFP, and C‐LFP batteries. d) CV curves of S‐LFP, R‐LFP, and C‐LFP batteries at a scan rate of 0.1 mV s^−1^. e) CV curves of the R‐LFP battery at varying scan rates. f) Linear fitting of peak current versus scan rate for the R‐LFP battery at different scan rates. g) GITT results for S‐LFP and R‐LFP. h) Comparison of regeneration performance between this work and previous studies. i) Voltage points for in situ EIS measurements over a charge–discharge cycle. j,k) EIS results for S‐LFP and R‐LFP batteries over a charge–discharge cycle. l,m) Fitted values of *R*_ct_ and ohmic resistance (*R*_o_) for S‐LFP and R‐LFP batteries.

Due to significant Li depletion, persistent Li–Fe antisite defects, and partial structural damage, S‐LFP batteries exhibit an initial discharge capacity of only 122 mAh g^−1^ at a rate of 0.1 C (where 1 C = 170 mAh g^−1^), which falls below the commercial discard threshold of 140 mAh g^−1^ (80% of maximum capacity). Moreover, at a rate of 1 C, S‐LFP maintains a persistently low capacity during cycling, making it unsuitable for reuse. Conversely, the R‐LFP battery regenerated using DES demonstrated an initial charge/discharge specific capacity of 155.6 mAh g^−^¹, exceeding the capacity of C‐LFP at 151 mAh g^−^¹ (see **Figure** **3**a), significantly boosting its maximum capacity performance.

To assess the rate performance before and after regeneration, specific capacities for S‐LFP at rates of 0.1 C, 0.5 C, 1 C, 2 C, and 5 C were recorded as 122.66, 115.42, 106.82, 93.3, and 72.44 mAh g^−1^, respectively. Post‐regeneration, R‐LFP exhibited substantial increases in specific capacities to 155.16, 141.52, 131.11, 118.52, and 95.6 mAh g^−1^ at the same respective rates (see Figure 3b). Notably, at a rate of 1 C, R‐LFP demonstrated a significant enhancement in cycling performance. Initially, S‐LFP had a capacity of 106.47 mAh g^−1^, retaining only 68% after 300 cycles. Post‐regeneration, R‐LFP maintained near‐maximal capacity at ≈130 mAh g^−1^ with a retention rate of 93% after 300 cycles, exhibiting superior cycling stability compared to C‐LFP (see Figure 3c). Additionally, remarkable improvements were observed under high‐rate cycling conditions at 5 C (Figure S18, Supporting Information). The notable increase in electrochemical performance can be attributed to the DES system's capability to precisely restore Li and provide a carbon source, enhancing conductivity and enabling R‐LFP to achieve excellent rate performance and cycling stability.

Further in‐depth analysis was performed to benchmark the electrochemical performance of R‐LFP against that of S‐LFP. The multifunctional properties of the DES were crucial in effectively repairing Li deficiencies, Li–Fe antisite defects, and structural degradation in S‐LFP. Figure 3d illustrates the oxidation and reduction potentials during the charge–discharge process at a scan rate of 0.1 mV s^−1^. In cyclic voltammetry (CV) testing, after the third cycle, the polarization voltage difference for R‐LFP was significantly reduced to just 0.15 V, compared to 0.42 V for S‐LFP, revealing enhanced reaction reversibility in R‐LFP following DES regeneration (Figure S19, Supporting Information). Figure 3e presents the CV curves for R‐LFP at scan rates ranging from 0.1 to 1 mV s^−1^, displaying distinct peak currents and voltages. The results obtained at different scan rates were examined using the Randles–Sevcik equation, highlighting the enhanced electrochemical kinetics post‐regeneration

(2) $$I_{p}=\left(2.65\;\;\times \;10^{5}\right)n^{\frac{3}{2}}{\mathrm{SD}}_{\mathrm{Li}}^{\frac{1}{2}}C_{\mathrm{Li}}v^{\frac{1}{2}}$$

In this equation, *I*_p_ denotes the peak current, *n* represents the number of electrons exchanged per molecule or ion during the redox process, *S* is the electrode surface region, *D*_Li_ refers to the diffusion coefficient of Li^+^, *C*_Li_ is the intensity of Li^+^ participating during the redox process, and *v* corresponds to the scan rate of the applied voltage. Constants such as *S*, *n*, and *C*_Li_ can be treated as invariant parameters. Consequently, the diffusion factor *D*_Li_ is inherently positive and exhibits a direct correlation with the slope of the *I*_p_/*v* ^1/2^ profile.^[^ ^36^, ^37^ ^]^ The R‐LFP demonstrates a more pronounced slope compared to the S‐LFP, indicating a higher Li^+^ diffusion (Figure 3f). This finding is consistent with the data obtained from the galvanostatic intermittent titration technique (GITT) displayed in Figure 3g. The slower Li^+^ migration in S‐LFP is ascribed to the presence of numerous Li–Fe antisite defects, which significantly obstruct Li^+^ transport. Consequently, higher activation energies are required throughout the charge–discharge process to overcome the resistance introduced by these defects. The enhanced Li^+^ diffusion capacity observed in R‐LFP highlights the multifunctional role of the DES in repairing Li–Fe antisites, thus improving the diffusion pathways and accelerating Li^+^ migration.^[^ ^38^, ^39^, ^40^ ^]^

These results show that regeneration using this Li‐containing DES yields excellent electrochemical performance. A comparison with other reported direct regeneration methods (see Table S2, Supporting Information) revealed that the DES used in this study achieved optimal repair efficacy and process conditions. Notably, the performance improvement of S‐LFP following DES treatment surpassed that of other methods. Furthermore, unlike many studies that require temperatures above 200 °C or high‐temperature calcination, our approach offers significant competitive advantages by directly regenerating spent LFP cathode material at a maximum reaction temperature of only 80 °C (Figure 3h).

In situ electrochemical impedance spectroscopy (EIS) was performed throughout a charge–discharge cycle to further elucidate the changes in internal impedance. The equivalent circuit model is detailed in Figure S24 of the Supporting Information. EIS measurements were captured at various voltages during the charge–discharge cycle (Figure 3i), with densely sampled points specifically during the plateau regions to more accurately determine impedance variations.^[^ ^41^ ^]^In the high‐frequency range (Figure 3j,k), a distinct semicircle emerges, reflecting charge transfer at the cathode interface. The diameter of this semicircle corresponds to the charge‐transfer resistance (*R*_ct_), and its left intercept indicates the ohmic resistance.^[^ ^42^ ^]^ In the low‐frequency range, the linear portion corresponds to the Warburg impedance, reflecting Li^+^ diffusion kinetics.

During the charging phase, *R*_ct_ progressively decreased, with a pronounced reduction observed in the plateau region (3.4–3.6 V), reaching its lowest at full charge (4.2 V). Conversely, *R*_ct_ exhibited a slight increase during discharge before stabilizing at a relatively consistent level.^[^ ^43^, ^44^ ^]^ Notably, R‐LFP showed a similar resistance trend to S‐LFP throughout the cycle but with significantly lower internal impedance across all voltages.

The analysis and fitting of the experimental EIS data, as presented in Tables S3 and S4 of the Supporting Information, indicated that the *R*_ct_ values for R‐LFP were substantially lower than those for S‐LFP during the charge–discharge cycles (Figure 3l). This enhancement in interfacial stability can be attributed to the dual functionality of the deep eutectic solvent (DES) during the regeneration process. Specifically, the DES supplies free Li⁺ ions that replenish lithium vacancies generated in S‐LFP during cycling, thereby restoring the material's stoichiometry and mitigating structural distortions or phase transitions associated with lithium deficiency. Concurrently, the DES facilitates the dissolution and removal of surface impurities from LiFePO₄—such as passivating residues—that otherwise hinder effective electrode–electrolyte contact. This synergistic effect of lithium replenishment and surface refinement is essential for re‐establishing a stable and efficient electrode interface (Figure 3m). Additionally, initial EIS measurements were performed on S‐LFP, R‐LFP, and C‐LFP and after 10 cycles. The impedance of R‐LFP was considerably lower than those observed with the other two battery types (Figure S23, Supporting Information), highlighting the effectiveness of the DES treatment in improving electrochemical performance and reducing impedance, thereby enhancing overall battery efficiency and longevity.

To assess the broader utility of this DES, the established process conditions were applied to the direct regeneration of spent Li cobalt oxide (S‐LCO) batteries. As showed in Figure S24 of the Supporting Information, the initial charge–discharge capacity at 0.1 C for S‐LCO increased from 100 to 128 mAh g^−1^. In contrast to the significant degradation observed in S‐LCO batteries after 200 cycles, the regenerated R‐LCO batteries maintained their initial capacity even after the same number of cycles, demonstrating enhanced cycling stability. The X‐ray diffraction (XRD) results shown in Figure S25 of the Supporting Information and the inductively coupled plasma (ICP) results in Figure S26 of the Supporting Information suggest that this DES facilitates the directional diffusion of Li^+^ into S‐LCO. Thus, beyond its efficient use in recovering and restoring spent LiFePO₄, this DES also exhibits potential for the direct regeneration of other spent Li‐ion battery cathode materials.

### 2.4 Microstructural Characterization of LFP before and after Regeneration Using DES

**Figure 4:**
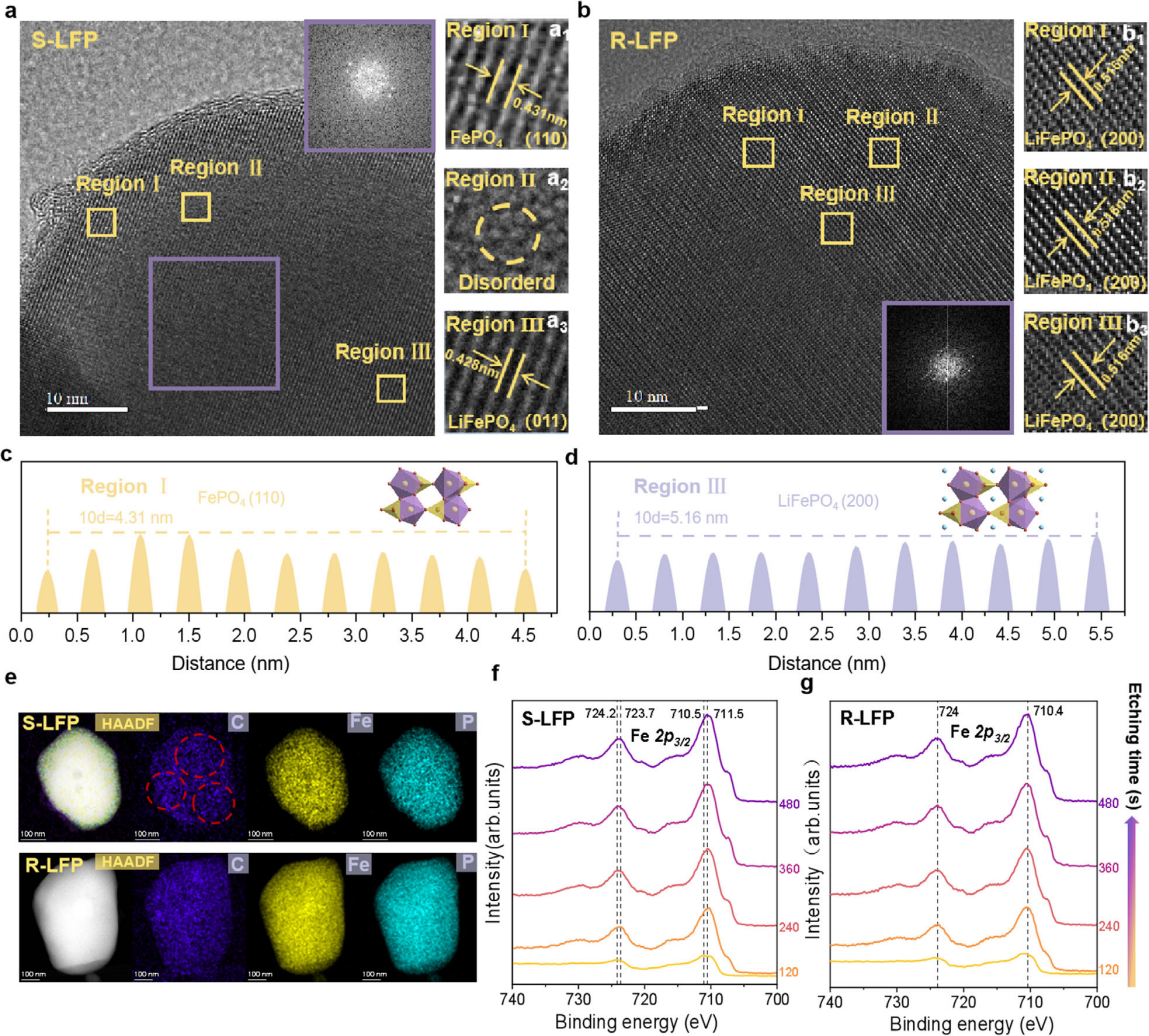
Microstructural characterization and phase transformation analysis of LFP. a) High‐resolution transmission electron microscopy (HRTEM) images of S‐LFP, with corresponding FFT patterns. (a_1_–a_3_) HRTEM images of specifically marked regions selected for analysis. b) HRTEM images of R‐LFP, with corresponding FFT patterns. (b_1_–b_3_) HRTEM images of specifically marked regions selected for analysis. c,d) Analysis and measurement of lattice spacings in S‐LFP and R‐LFP. e) High‐angle annular dark‐field scanning transmission electron microscopy (HAADF‐STEM) images and EDS maps of S‐LFP and R‐LFP. f,g) Fe 2*p* depth‐profiled XPS images of S‐LFP and R‐LFP.

To preliminarily investigate the surface morphology, scanning electron microscopy (SEM) was utilized, revealing adhesive residues on S‐LFP absent in the DES‐regenerated R‐LFP (Figure S27, Supporting Information). For a deeper analysis of phase composition and microstructure before and after DES treatment, transmission electron microscopy (TEM) captured images of distinct regions (Figure 4a). Three representative regions were selected for further analysis. First, Region I (Figure 4a1) displayed a narrow, irregular lattice spacing at the edge of S‐LFP, where the measurement of ten diffraction peaks yielded a lattice spacing of 0.431 nm, corresponding to the 110 plane of FePO₄ (Figure 4c). Second, Region II (Figure 4a2) revealed twisted, blurry stripes toward the core of S‐LFP. Fast Fourier transform (FFT) analysis in this region indicated a disordered diffraction pattern, reflecting a chaotic phase mixture of degraded FePO₄ and residual LiFePO₄.^[^ ^9^, ^39^ ^]^ Lastly, Region III (Figure 4a3) at the core of S‐LFP displayed wider lattice fringes with a spacing of 0.428 nm, consistent with the (011) plane of LiFePO₄. These findings suggest that Li loss predominantly occurs at the particle surface and edges, whereas the TEM image of DES‐treated R‐LFP (Figure 4b) shows uniformly spaced lattice fringes. The three areas with the highest contrast were selected for detailed analysis, and measurements of 10 diffraction peaks indicated a consistent lattice spacing of 0.516 nm, corresponding to the (200) plane of LiFePO₄ (Figure 4d). The FFT pattern of R‐LFP displayed well‐ordered, regular diffraction spots, signifying that the DES treatment resulted in a uniform single‐phase LiFePO₄ crystal structure. This is attributed to the selective diffusion of Li^+^ from DES into vacant sites of S‐LFP, driving the conversion of degraded FePO₄ phases and disordered mixed phases back into a homogeneous LiFePO₄ structure, thus regenerating R‐LFP. Energy dispersive spectroscopy (EDS) mapping of the elemental distribution in R‐LFP (Figure 4e) revealed a uniform distribution of Fe, P, and O. Additionally, from the EDS mapping of carbon, we observed that the carbon distribution in S‐LFP appeared uneven and exhibited pronounced particle aggregation. After low‐temperature regeneration using DES, the carbon layer became noticeably more uniform and continuous, which is consistent with the increased specific surface area revealed by the BET analysis (Figure S30, Supporting Information). The improved uniformity and integrity of the carbon coating in R‐LFP, facilitated by the DES treatment, significantly enhanced its electrical conductivity, increasing from 10^−⁴^ to 10^−^^2^ S cm^−1^ (Figures S28 and S29, Supporting Information).

Additionally, depth‐profiling X‐ray photoelectron spectroscopy (XPS) provided insights into the valence states of Fe from the surface to the interior. On the surface of S‐LFP, the Fe 2*p*_₃/₂_ peak at 711.5 eV confirmed the presence of surface Fe(III). As the etching depth increased, the peak shifted to 710.5 eV, indicating that most of the Fe within S‐LFP was in the Fe(II) state (Figure 4f), consistent with the TEM results. After DES regeneration, R‐LFP displayed a stable Fe 2*p*_₃/₂_ peak at 710.5 eV from surface to core, with Fe(III) completely eliminated (Figure 4g), confirming that the DES treatment successfully converted degraded FePO₄ back into uniform LiFePO₄. Under optimized regeneration conditions using our LiCl–C₂H₆O₂ DES, the process achieved Li supplementation, Fe^3+^ phase to Fe^2+^ phase conversion, and repair of Li–Fe antisite defects. This regeneration relies on the synergistic effects of the DES components to effectively restore both the chemical composition and the structure of S‐LFP.

### 2.5 Mechanistic Analysis of Direct Regeneration Process of S‐LFP Using DES

Through the preceding investigations and discussions, the LiCl–C₂H₆O₂ DES has demonstrated a multifunctional synergistic effect in the regeneration process of S‐LFP. This process can be summarized as follows

(3) $$\mathrm{FeP}O_{4}+Li^{+}+e^{-}\to \mathrm{LiFeP}O_{4}$$

**Figure 5:**
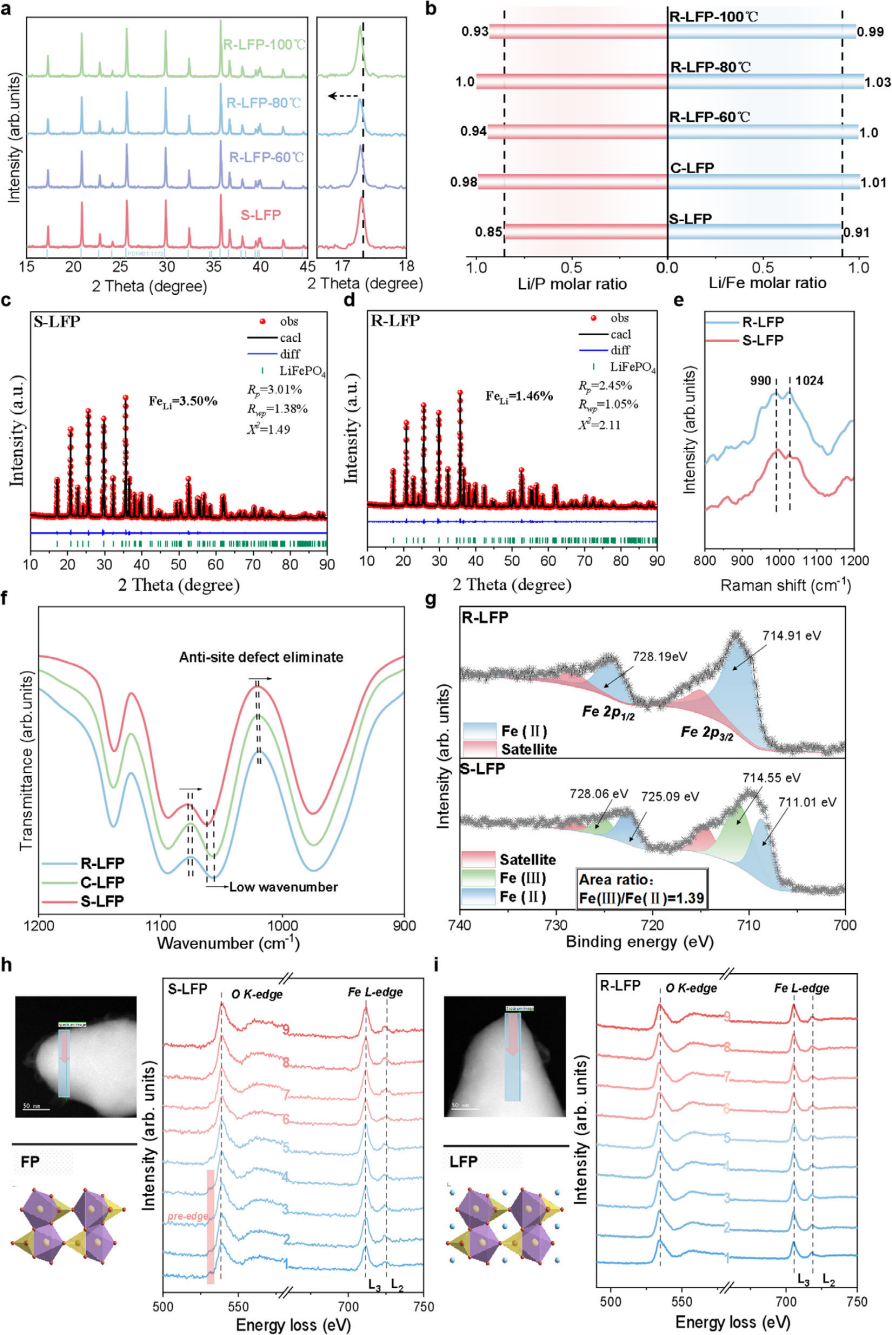
Mechanistic analysis of DES‐regenerated S‐LFP. a) XRD patterns of S‐LFP and R‐LFP regenerated at various temperatures. b) Li/P and Li/Fe molar ratios for S‐LFP and R‐LFP regenerated at different temperatures based on inductively coupled plasma‐optical emission spectroscopy (ICP‐OES) analysis. c,d) XRD and the XRD refinement results of S‐LFP and R‐LFP. e) Raman spectra of S‐LFP and R‐LFP. f) FT‐IR spectra of S‐LFP and R‐LFP. g) The XPS results of Fe 2*p* for S‐LFP and R‐LFP. h) HAADF‐STEM image of S‐LFP with EELS spectra showing the *O‐K* edge and Fe–*L* edges. i) HAADF‐STEM image of R‐LFP with EELS spectra showing the *O‐K* and Fe–*L* edges.

The structural and compositional analyses presented earlier effectively illustrated the phase transformation process. To further analyze the phase composition of LFP before and after regeneration, XRD was conducted (**Figure** **5**a). The XRD pattern of S‐LFP revealed no diffraction peaks corresponding to the degraded FePO₄ phase, likely due to its low proportion and the partial degradation of S‐LFP. Upon magnifying specific peaks, a slight shift in the (200) peak to lower angles was observed for R‐LFP with increasing repair temperature. According to Bragg's law (2*d*sin*θ* = *nλ*), a decrease in *θ* (with a constant wavelength *λ*) implies an increase in lattice spacing *d*. We hypothesize that this phenomenon is related to the electrostatic repulsion generated within the lattice as Li^+^ occupies vacancies in the S‐LFP and the conversion of Fe^3+^ to Fe^2+^.^[^ ^45^ ^]^ This result aligns with previous TEM findings and closely corresponds to the (200) crystal plane peak position of C‐LFP (Figure S31, Supporting Information). Additionally, ICP results indicate that the molar ratios of Li/Fe and Li/P in S‐LFP were only 0.85 and 0.91, respectively, evidencing a Li‐deficient state. After low‐temperature DES treatment, the Li/Fe and Li/P molar ratios in R‐LFP at 80 °C reached 1.03 and 1.0, meeting theoretical values and surpassing those of C‐LFP (Figure 5b). Compared to the regeneration performance at 60 °C, the reaction conditions at 80 °C provide sufficient thermal energy to facilitate the diffusion of Li⁺ into lithium‐deficient sites within the S‐LFP structure. However, when the temperature is increased to 100 °C, the lithium replenishment becomes less effective. This decline is attributed to partial decomposition of the LFP lattice at elevated temperatures, which leads to the deintercalation of Li⁺ from the crystal structure. In practical LFP materials, the Li/Fe ratio typically exceeds the theoretical value of 1.0. Therefore, we conclude that under hydrothermal conditions at 80 °C, the DES exhibits the most effective lithium replenishment capability. The insertion of Li ions leads to an increase in interplanar spacing of certain crystal facets, enhancing ionic conductivity and electrochemical performance.

These findings illustrate the role of DES in Li supplementation and phase transformation within S‐LFP; however, the repair of Li–Fe antisite defects warrants further attention. To overcome the activation energy barrier of phase transitions, the hydroxyl group in ethylene glycol serves as an electron donor, providing additional electrons to reduce Fe^3^⁺ to Fe^2^⁺. During this reduction process, Li^+^ ions in the DES selectively diffuse into lattice vacancies within FePO₄, guiding the relocation of Fe ions occupying Li sites return to their intrinsic octahedral sites. Li ions tend to diffuse along the [010] direction, suggesting this is the primary diffusion pathway.^[^ ^15^, ^16^, ^46^ ^]^ By effectively addressing the Li–Fe antisite defects in S‐LFP, the Li‐ion diffusion environment is improved, thereby restoring the electrochemical performance of LFP. High‐resolution XRD refinement before and after regeneration (Figure 5c,d) confirms that both S‐LFP and R‐LFP possessed the same space group. Tables S6–S10 of the Supporting Information provide detailed information on the Fe‐occupying Li sites in the S‐LFP, C‐LFP, and R‐LFP samples regenerated at different temperatures. The proportion of Fe–Li antisite defects in the S‐LFP composition was ≈3.50%. After regeneration with DES at 80 °C, the Li–Fe antisite defects in R‐LFP were reduced to a minimum of 1.46% (Figures S32–S34, Supporting Information). FT‐IR analysis further corroborated this result, where characteristic transmission peaks of LFP, associated with P–O stretching modes in phosphate tetrahedra, appeared at 1050 and 1000 cm^−1^. In R‐LFP, these peaks shifted to lower wavenumbers than in S‐LFP, indicating a reduction or elimination of Li–Fe antisite defects (Figure 5f).^[^ ^47^, ^48^ ^]^ Raman spectroscopy (Figure 5e) also revealed that the disappearance of Li–Fe defects in R‐LFP leads to symmetric P─O bond stretching, resulting in symmetric peaks at 990 and 1024 cm^−19^.

X‐ray photoelectron spectroscopy (XPS) was employed to elucidate the Fe valence state at the LFP particle surface (Figure 5g). In the degraded S‐LFP, two principal binding energy peaks were observed at 714.55 and 711.01 eV, which corresponded to Fe^3^⁺ and Fe^2^⁺ in the Fe 2*p*_₃/₂_ spectrum, respectively. Additional, two principal binding energy peaks were observed at 728.06 and 725.09 eV, which corresponded to Fe^3^⁺ and Fe^2^⁺ in the Fe 2*p*_₁/₂_ spectrum, respectively. These suggest that FePO₄ is the predominant surface phase in S‐LFP. After treatment with DES, the prominent binding energy peaks in the regenerated R‐LFP shifted to 728.19 and 714.9 eV, corresponding to Fe^2^⁺ in the Fe 2*p*_₁/₂_ and Fe 2*p*_₃/₂_, spectra, respectively. The absence of Fe^3^⁺ in the regenerated sample confirmed that DES facilitated the complete reduction of Fe^3^⁺ to Fe^2^⁺.^[^ ^49^, ^50^ ^]^ Moreover, during the regeneration process, the reinsertion of Li⁺ into the crystal lattice alters the local electronic environment of Fe^2^⁺ through Li–O–Fe coupling. The introduction of Li⁺ draws the electron cloud of neighboring O^2−^ ions toward itself, thereby indirectly weakening the electron shielding effect experienced by Fe^2^⁺. This charge redistribution effectively increases the effective nuclear charge of Fe^2^⁺, resulting in a shift toward higher binding energy in the XPS spectra. This observation further supports the successful lithium replenishment facilitated by the DES treatment, in agreement with the previously discussed ICP and XRD results.^[^ ^51^, ^52^ ^]^

Three key functions of the LiCl–C₂H₆O₂ DES contribute to the effective regeneration of S‐LFP. 1) DES serves as a direct Li source, replenishing Li in S‐LFP at low temperatures and restoring its original chemical composition. This process is nonspontaneous, with ethylene glycol, an HBD in DES, playing a critical role by donating electrons through its hydroxyl groups, enabling the reaction to proceed spontaneously at 80 °C. 2) The reducing atmosphere created by ethylene glycol stabilizes Fe^2^⁺ during regeneration, preventing its reoxidation to Fe^3^⁺ and promoting the reduction of Fe^3^⁺ back to Fe^2^⁺. 3) The reducing environment decreases the initiation barrier for Fe ions to vacate Li sites and return to their native octahedral positions, thereby mitigating Li–Fe antisite defects and enhancing the Li‐ion diffusion pathway.

While these findings highlight that Li deficiency and Fe^3^⁺ formation are mainly concentrated on the S‐LFP particle surface, further analysis of individual particles is necessary to fully comprehend the mechanisms behind S‐LFP degradation. Electron energy loss spectroscopy (EELS) was conducted on single particles to evaluate the elemental valence states and distributions. The changes in Fe valence states were accurately tracked via energy loss at the Fe *L*₂ and *L*₃ edges. High‐angle annular dark‐field scanning TEM (HAADF‐STEM) images (shown in Figure 5h,i) depict the scan region and direction, with the results presented on the right. The Fe *L*₂ and *L*₃ edges were characterized by two distinct white lines due to 2*p* spin–orbit splitting into unoccupied 3*d* orbitals.^[^ ^50^, ^53^ ^]^ The energy loss at these edges indicates changes in the Fe valence states. An *O‐K* edge arising from Fe excitation from the 1*s* orbital into unoccupied O 2*p* orbitals was notable; Fe^3^⁺ phosphates exhibited a distinct pre‐edge feature at the *O‐K* edge,^[^ ^54^ ^]^ whereas Fe^2^⁺ phosphates lacked this feature. This phenomenon originates from the presence of Fe^3^⁺, whose 3*d*⁵ orbital holes enhance the electron transition from O 2*p* to Fe 3*d*, leading to an increased pre‐edge peak intensity at the *O‐K* edge in the EELS spectrum. This enhancement provides compelling evidence for the formation of a FePO₄ phase on the surface of S‐LFP. Additionally, energy loss at the Fe *L*₂ edge (points 1–10) exhibits varying degrees of shift. In contrast, the *L*₃ edge remains unchanged, which can be attributed to the selective electronic rearrangement associated with low‐spin Fe^3^⁺ (t₂g⁵e_g⁰). In a strong octahedral crystal field, the fully occupied t₂g orbitals (t₂g⁵) enhance spin–orbit coupling for the 2*p*_₁/₂_ → 3d(t₂g) transition, resulting in a noticeable shift at the *L*₂ edge. Meanwhile, the unoccupied e_g orbitals maintain the stability of the 2*p*_₃/₂_ → 3d(e_g) dominated *L*₃ edge. This behavior also reflects a gradual transition from Fe^2^⁺ to Fe^3^⁺ from the surface to the core of the particle, indicating the coexistence of a mixed FePO₄/LiFePO₄ phase.^[^ ^55^, ^56^ ^]^ The *O‐K* edge showed a similar trend, suggesting that the surface consists of FePO₄, with LiFePO₄ distributed toward the edge. This aligns with the prior XPS depth‐profiling results (Figure 4f,g), demonstrating that S‐LFP degradation is concentrated at the cathode surface.

Compared to S‐LFP, no *O‐K* pre‐edge peaks were observed in the regenerated R‐LFP, and the energy losses at the Fe *L*₂ and *L*₃ edges were lower, at 723.6 and 711.5 eV, respectively. This confirms that the regenerated R‐LFP is a homogeneous Fe^2^⁺ phase (Figure 5i), indicating that the Li deficiency was compensated, Fe^3^⁺ was eliminated, and Li–Fe antisite defects were corrected. The multifunctional DES played a crucial role in achieving these transformations.

### 2.6 Technoeconomic Analysis

**Figure 6:**
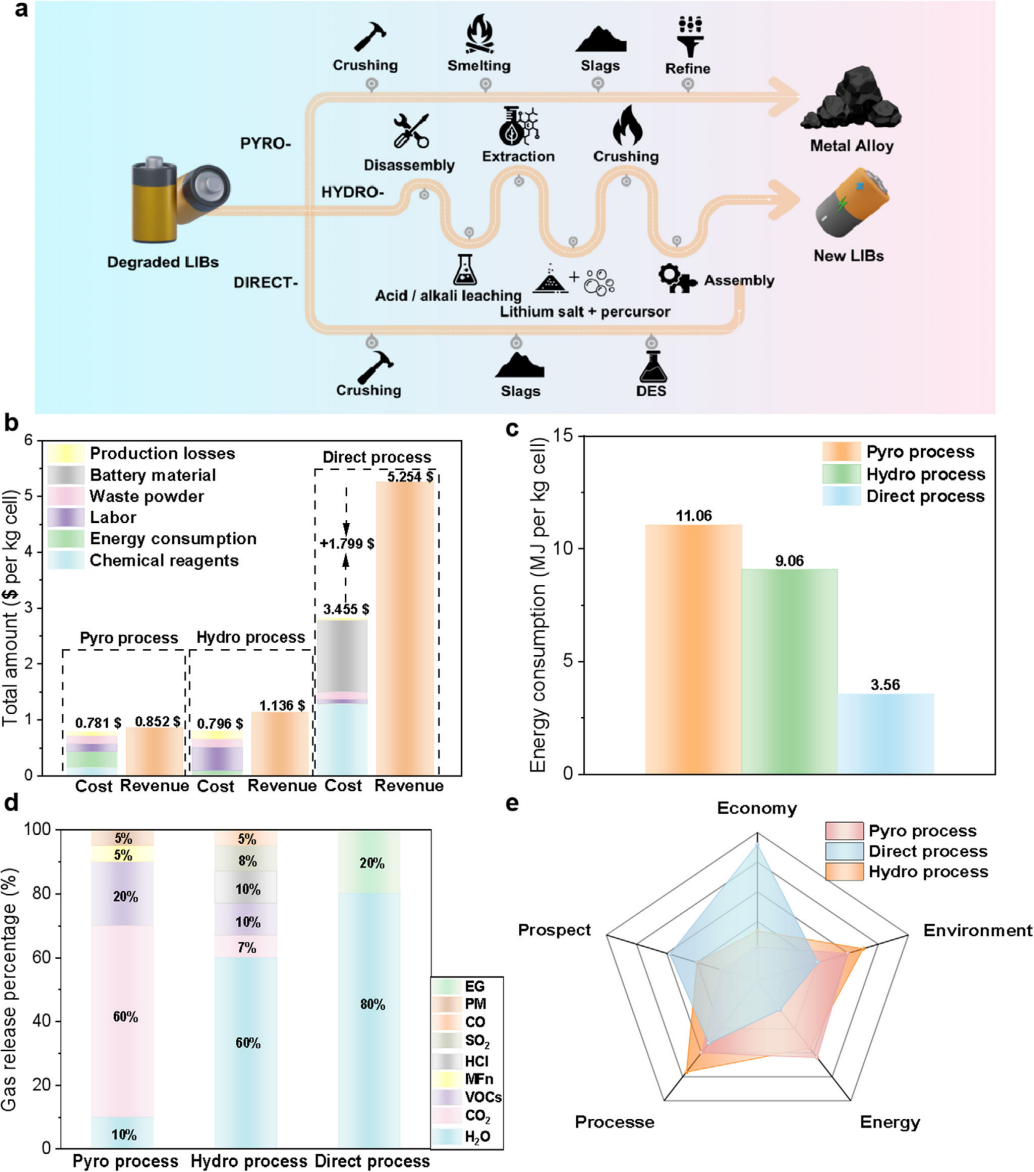
Technoeconomic analysis of different LFP battery recycling technologies. a) Process flow diagram of various LFP battery recycling technologies. b) Cost and profit analysis of different LFP battery recycling methods. c) Energy consumption analysis for different LFP battery recycling technologies. d) Emissions and their proportions for various LFP battery recycling methods. e) Comprehensive evaluation chart of different LFP battery recycling technologies.

**Figure** **6**a illustrates the current mainstream methods for recycling spent Li iron phosphate (LFP) batteries, including pyrometallurgical, hydrometallurgical, and direct regeneration methods proposed in this study. The direct regeneration approach significantly reduces the number of processing steps, notably omitting high‐temperature calcination and acid–base leaching. This reduction streamlines the process, decreasing energy consumption, and lessening the environmental impact due to reduced emissions of waste gases and liquids. Specifically, the energy consumption of the DES direct regeneration process is significantly lower at 3.56 MJ kg^−1^ compared to 11.06 MJ kg^−1^ for pyrometallurgy and 9.06 MJ kg^−1^ for hydrometallurgy (see Figure 6c). Moreover, the complex waste treatment required for handling toxic byproducts such as CO, SO_2_, HCl, and PM2.5 in pyrometallurgical and hydrometallurgical methods adds to operational complexity and costs (see Figure 6d; Figures S37–S39 and Table S14, Supporting Information). In contrast, the direct regeneration process in this study mainly produces water and ethylene glycol vapors as emissions, aligning with the green, environmentally friendly ethos of DES usage. Consequently, the DES‐based regeneration process offers clear advantages regarding energy savings and emission reduction.

Economic simulations using the EverBatt 2023 model estimated the profitability of each recycling method (Tables S11 and S12, Supporting Information). The pyrometallurgical approach yielded the lowest profit at $0.069 per kg, followed by hydrometallurgy at $0.341 per kg. The direct regeneration method, however, showed significantly higher profitability, reaching $1.799 per kg (see Figure 6b). We also compared our regeneration process with those reported in previous studies. As shown in Figure S35 of the Supporting Information, our method still demonstrates a clear advantage in terms of profit generated per kilogram of regenerated material. To maximize this profit, the cost‐efficiency of the reagents was a key consideration during the selection process, leading to the choice of low‐cost ethylene glycol and Li chloride as DES components. The repeated recycling of the DES reagent was validated for effective reuse in the direct regeneration of spent LFP, further lowering reagent costs and enhancing profitability. Although the model calculations may not be entirely precise, they provide reasonable guidance for selecting recycling methods.

Globally, LFP battery recycling is still nascent, with suboptimal recycling rates contributing to resource depletion and environmental pollution. As evidenced in Figure 6e, through comparison with conventional regeneration methods, we demonstrate that this DES‐based direct regeneration approach offers a more economically viable, environmentally sustainable, and technologically promising recycling pathway for S‐LFP cathodes. Further refinements in the use and scalability of this low‐cost and reusable DES reagent are warranted, with pilot‐scale studies recommended to enhance its future economic feasibility. Ultimately, this approach holds promise for meeting large‐scale standards for the direct regeneration of spent LFP batteries.

## 3 Conclusion

In summary, we utilized a green, multifunctional, Li‐containing DES to directly regenerate S‐LFP cathodes. This DES Linot replenishes Li in S‐LFP and creates a reducing environment that facilitates the conversion of degraded Fe^3+^ phase to Fe^2+^ phase and repairs the Li–Fe antisite defects. To evaluate the effectiveness of this regeneration approach, we conducted a comprehensive analysis using various characterization techniques and electrochemical measurements. The XRD and ICP results showed that the soluble Li salts in the DES selectively migrated to Li‐deficient vacancies within S‐LFP while also restoring the spent FePO_4_ phase. Refinement of XRD results and FT‐IR analysis confirmed the effective reduction of Li–Fe antisite defects in S‐LFP, enhancing Li^+^ diffusion. The R‐LFP exhibited excellent rate capability, high capacity retention, and cycling stability following the regeneration process. Compared to traditional recycling methods, such as pyrometallurgical and hydrometallurgical processes, and even commonly used hydrothermal methods, this low‐cost, readily available Li‐containing DES enables direct regeneration of S‐LFP at a low temperature of 80 °C. This avoids complex high‐temperature calcination processes and yields greater economic benefits. As a novel, environmentally friendly solvent, the DES‐based direct regeneration process supports environmental sustainability and offers potential for reuse, promoting economic and ecological viability. Additionally, the S‐LFP used in this study was sourced directly from industrial waste powders, paving the way for the large‐scale direct regeneration of S‐LFP cathode materials for practical applications.

## 4 Experimental Section

### Materials

Major chemical reagents used in this study included ethylene glycol (C_2_H_6_O_2_, AR, ≥ 99%), purchased from Xilong Chemical Co., Ltd.; Li chloride (LiCl, AR, ≥99%) from Titan Technology Co., Ltd.; *N*‐methylpyrrolidone (NMP, GC, ≥99%); commercial LFP cathode powder (C‐LFP); 1 m LiPF_6_ in ethylene carbonate (EC):dimethyl carbonate (DMC):diethyl carbonate (DEC) in a 1:1:1 volume ratio; polyvinylidene fluoride (PVDF, 5130); conductive carbon black (Super P Li); polypropylene membrane (Celgard 2500); Al foil; Li metal foil; and C‐LFP powder, all purchased from Kelude New Energy Technology Co., Ltd. The spent cathode powder (S‐LFP) was provided by Tafier New Energy Technology Co., Ltd.

### Battery Disassembly and S‐LFP Cathode Material Separation

After electrolyte cycling to a maximum capacity below 80% of its theoretical value, the original pouch cell was designated as scrap. After removing the Al plastic film, the cathode (Al foil), anode (Cu foil), and polypropylene membrane were manually separated. The required cathode material, evenly coated on both sides of the Al foil, was placed in a tube furnace and calcined at 550 °C for 3 h with a heating rate of 5 °C min^−1^. During this process, the binder (PVDF) in the cathode material underwent carbonization, which allowed the active material to separate naturally from the Al foil. Finally, the collected cathode material was dried overnight in a vacuum drying oven at 70 °C to ensure stability. The final cathode material, designated as S‐LFP, was used in subsequent experiments.

### Direct Regeneration Process

1) Ethylene glycol (32.56 g) and Li chloride (7.44 g) were added in a 3:1 ratio into a glass vial with a magnetic stirrer and then placed in an oil bath at 100 °C. After stirring for 20 min, the solid was transformed into a transparent viscous solution, forming the DES. 2) The DES and S‐LFP were mixed in a solid‐to‐liquid ratio of 20:1 to ensure efficient contact between the reactants. The mixture was then subjected to a 12 h reaction under magnetic stirring and oil bath heating. Temperature gradients (60 °C, 80 °C, 100 °C, and higher) were used to explore the effects of temperature on the regeneration process and product properties. After stirring, the mixture was filtered, and the R‐LFP cathode material was washed multiple times with distilled water to remove impurities and then dried for 12 h in a 70 °C thermostatic oven to ensure complete drying and stable performance. The regenerated R‐LFP required no annealing treatment and was thus ready for further testing.

### Electrochemical Performance Testing

To assess the electrochemical performance of S‐LFP, R‐LFP, and C‐LFP, CR2032 coin cells were fabricated. The cathode slurry was prepared by blending the active material, poly(vinylidene fluoride) (PVDF) binder, and carbon black (conductive additive) in an 80:10:10 mass ratio, followed by addition of *N*‐methyl‐2‐pyrrolidone (NMP) as a dispersant to achieve a solid content of 38–40%. The slurry was homogenized in a centrifuge at 600 rpm for 6 min. A uniform coating of 10 µm was applied to Al foil, dried at 80 °C in an air oven for 2 h, then transferred to a vacuum oven at 80 °C overnight. The dried cathode sheets were cut into circular cathodes with diameters of 13 mm. Assembly was conducted in an argon‐filled glove box (H_2_O < 0.01 ppm, O_2_ <0.01 ppm), with Li metal disks (15 mm diameter, 0.45 mm thickness) used as anodes, 40 µL of 1 m LiPF_6_ EC:DEC:DMC (1:1:1) solution as the electrolyte, and Celgard 2500 as the separator, forming the CR2032 coin cells. Cells were stabilized in a 25 °C incubator for 8 h to ensure electrolyte infiltration. Long‐term cycling performance was tested at a 1 C rate (1 C = 170 mAh g^−1^) within a 2.5–4.2 V voltage range, while rate capability was tested at 0.1 C, 0.5 C, 1 C, 2 C, and 5 C (1 C = 170 mAh g^−1^) under 25 °C conditions. CV and EIS measurements were performed using a multichannel electrochemical workstation (VSP‐3e) at 25 °C. CV measurements were performed over a 2.5–4.2 V range at scan rates of 0.1, 0.2, 0.4, 0.6, 0.8, and 1 mV s^−1^. EIS measurements were conducted at a 0.1 C current density over a 2.5–4.2 V range, with frequencies ranging from 10 mHz to 100 kHz.

### Characterization

The solution properties were characterized using FT‐IR (Nicolet iS50), while TG‐DSC (Mettler TGA2) was used to analyze the solid and liquid mass changes with temperature. ICP‐OES (Arcos II MV) was used to determine the compositions of the cathode materials. XRD (D8 Advance 250) with Cu Kα radiation characterized the crystal structure of the powders, with 2*θ* scans from 10° to 90°. In situ XRD experiments using half‐cells covered the 10°–50° scan range, with a voltage range of 3–4.2 V and a current density of 0.1 C (1 C = 274 mAh g^−1^). XPS with Thermo Fisher ESCALAB Xi^+^ and Al K*α* radiation (*hv* = 1486.6 eV) analyzed elemental distributions, with binding energies calibrated to the standard C 1*s* peak at 284.8 eV. SEM (SU8010) and TEM (FEI Tecnai G2 F30) were used to examine the sample morphology. Raman spectroscopy (Horiba LabRAM HR Evolution) was used to characterize the material structure, and EELS (Thermo Fisher Spectra 300) was used to measure the oxidation state distributions of single LFP grain.

## Conflict of Interest

The authors declare no conflict of interest.

## Supporting information



Supporting Information

## Data Availability

The data that support the findings of this study are available from the corresponding author upon reasonable request.
