# Supplementary material for: Direct and Low‐Temperature Regeneration of Degraded LiFePO₄ Cathodes at Ambient Conditions Using Green and Sustainable Deep Eutectic Solvent
Source: Adv Sci (Weinh). 2025 May 20;12(28):2504683. doi: 10.1002/advs.202504683 (PMC12302551; doi:10.1002/advs.202504683)
Supplement: Supplementary file 1 — Supporting Information [file ADVS-12-2504683-s001.docx]

**Supporting Information**

**Direct and Low-Temperature Regeneration of Degraded LiFePO₄ Cathodes at Ambient Conditions Using Green and Sustainable Deep Eutectic Solvent**

*Yixin Lin, ^b, c, d #^ Tiansheng Wang, ^*a, b, c, d, e #^ Chaochao Gao, ^b, c, d, e^ Xiaoxuan Zhang, ^a^ Wen Yu, ^b, c, d^ Mi Wang, ^b, c, d^ Chao Yang, ^*a^ Jiaheng Zhang, ^*b, c, d, e, f^*

^a^School of Science, Harbin Institute of Technology (Shenzhen), Shenzhen, 518055, China;

^b^School of Materials Science and Engineering, Harbin Institute of Technology (Shenzhen), Shenzhen, 518055, China;

^c^Research Centre of Printed Flexible Electronics, Harbin Institute of Technology (Shenzhen), Shenzhen, 518055, China;

^d^Sauvage Laboratory for Smart Materials, School of Materials Science and Engineering, Harbin Institute of Technology (Shenzhen), Shenzhen, 518055, China;

^e^Shenzhen Shinehigh Innovation technology Ltd., Taoyuan Street, Nanshan District, Shenzhen 518055, China

^f^Department of Chemistry, University of Idaho, Moscow, Idaho 83844-2343, USA.

^#^These authors contributed equally to this work.

**Corresponding author**

*e-mail: [wangtiansheng@hit.edu.cn](mailto:wangtiansheng@hit.edu.cn) (Tiansheng Wang); [xyyang@hit.edu.cn (Chao](mailto:xyyang@hit.edu.cn%20(Chao) Yang); [zhangjiaheng@hit.edu.cn](mailto:zhangjiaheng@hit.edu.cn) (Jiaheng Zhang)


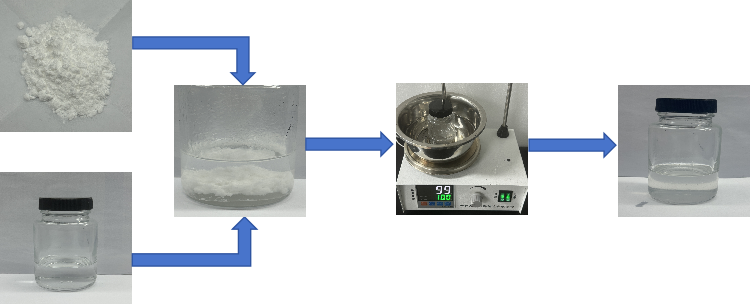


Cooling

100 ^o^C/0.5h

LiCl

C_2_H_6_O_2_

DES

**Supplementary Fig. S1** Synthesis process of Deep Eutectic Solvents (DES).


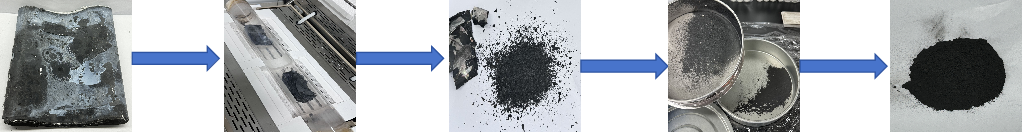


550 ^o^C/3h

Separate

S-LFP

Screen

Cathode plate

**Supplementary Fig. S2** Process for recovering waste powder (S-LFP).


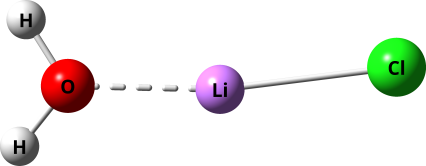

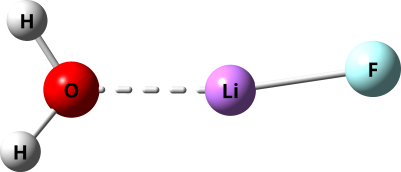

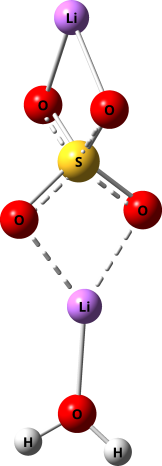


H_2_O-LiSO_4_

H_2_O-LiF

H_2_O-LiCl


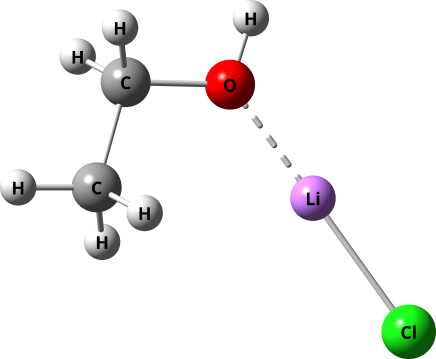

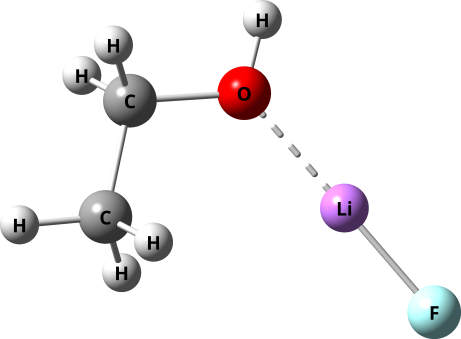

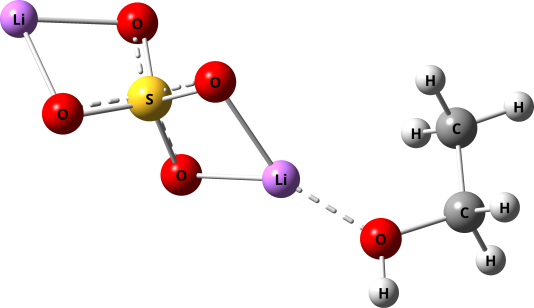


C_2_H_5_O-LiSO_4_

C_2_H_5_O-LiCl

C_2_H_5_O-LiF


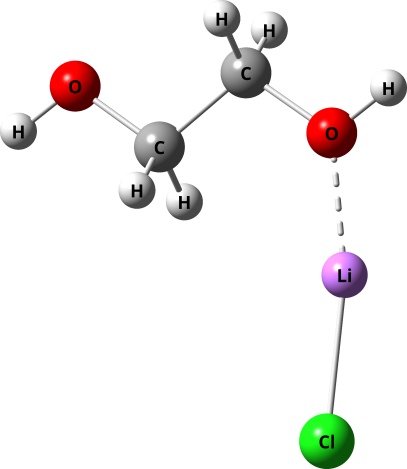

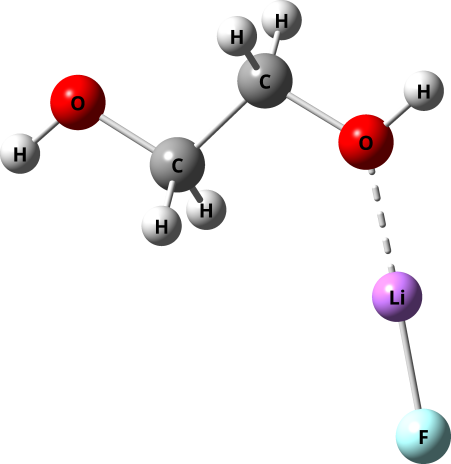

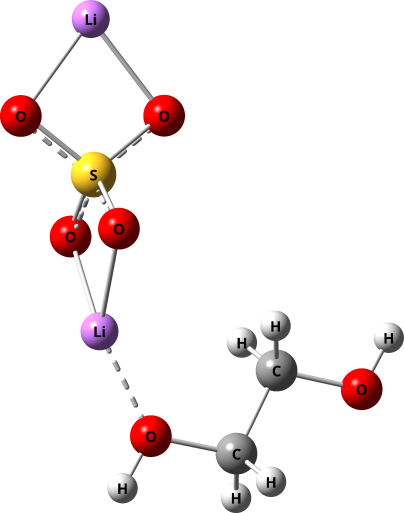


C_2_H_6_O_2_-LiSO_4_

C_2_H_6_O_2_-LiF

C_2_H_6_O_2_-LiCl


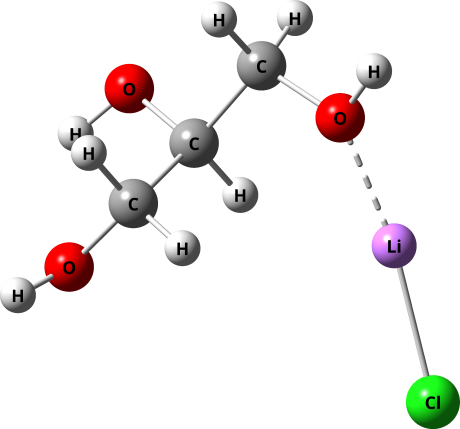

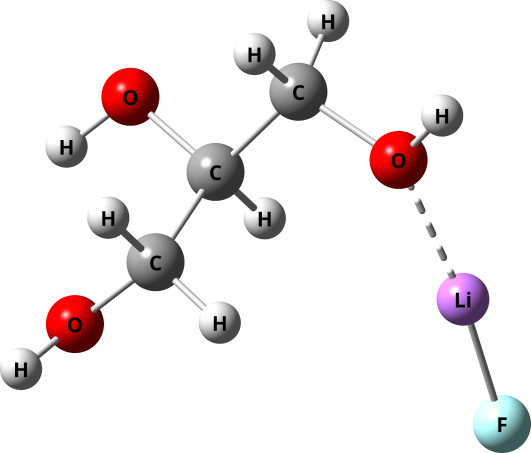

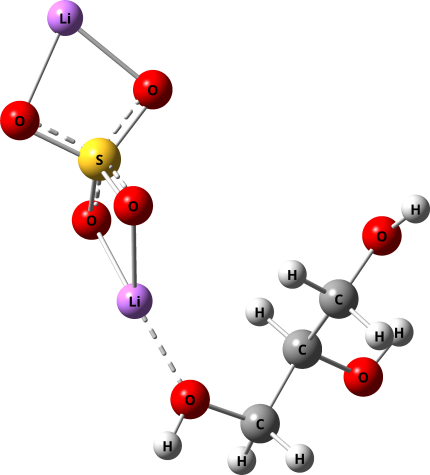


C_3_H_8_O_3_-LiSO_4_

C_3_H_8_O_3_-LiF

C_3_H_8_O_3_-LiCl

**Supplementary Fig. S3** Intermolecular interaction energies between different lithium salts and HBDs


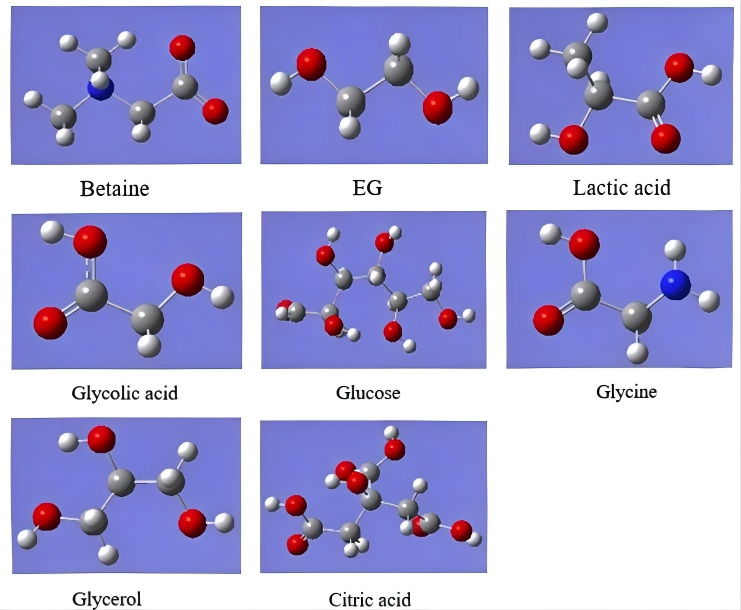


**Supplementary Fig. S4** Model construction for HOMO-LUMO calculations.


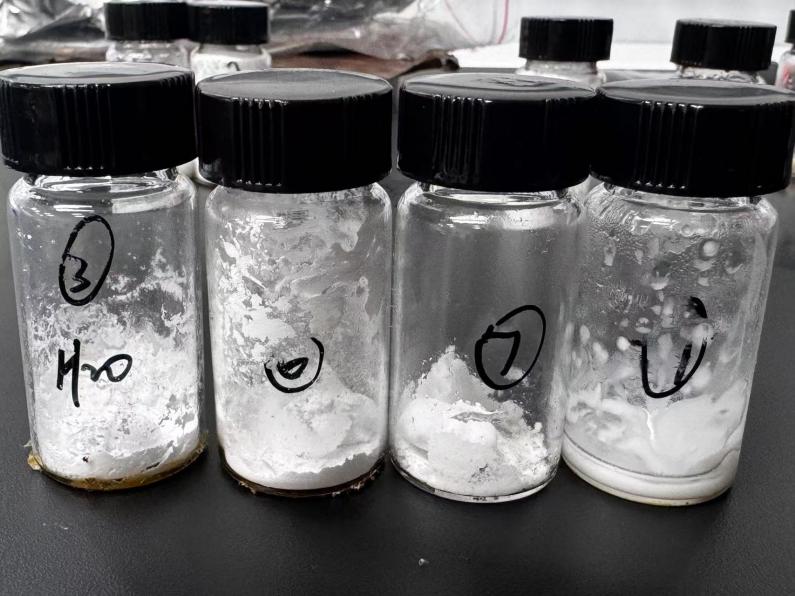


LiSO_4_ - H_2_O, EtOH, EG, GLY


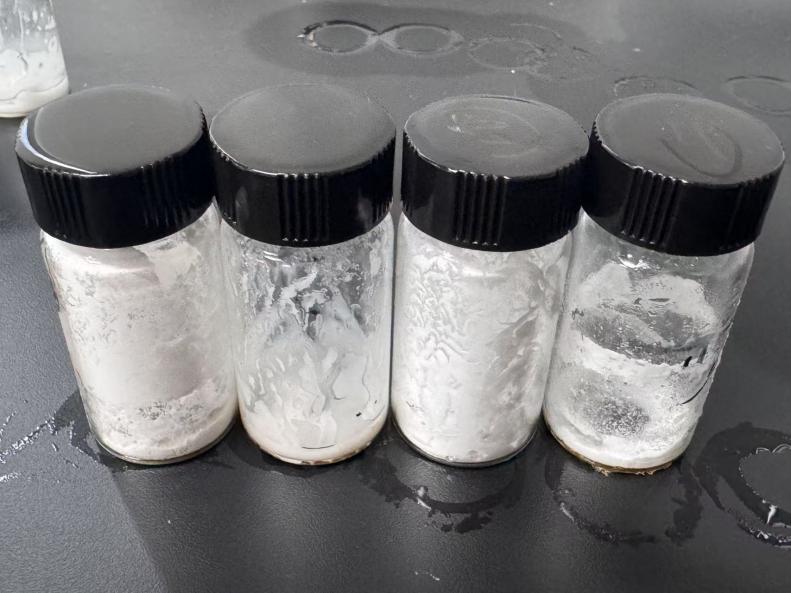


LiF -H_2_O, EtOH, EG, GLY


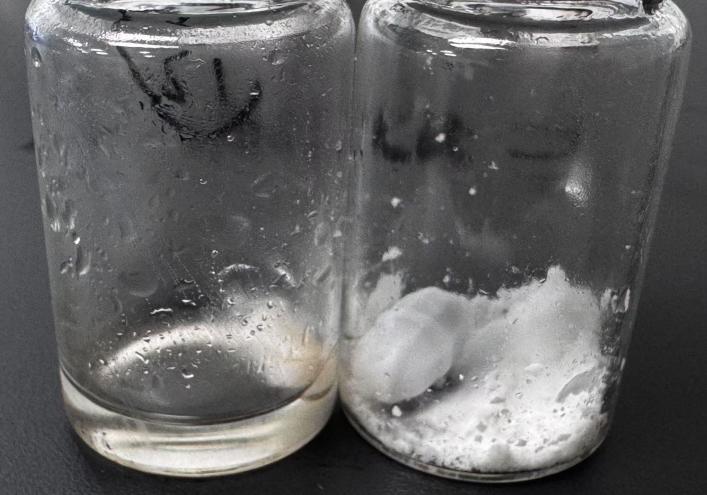


LiCl -H_2_O, EtOH


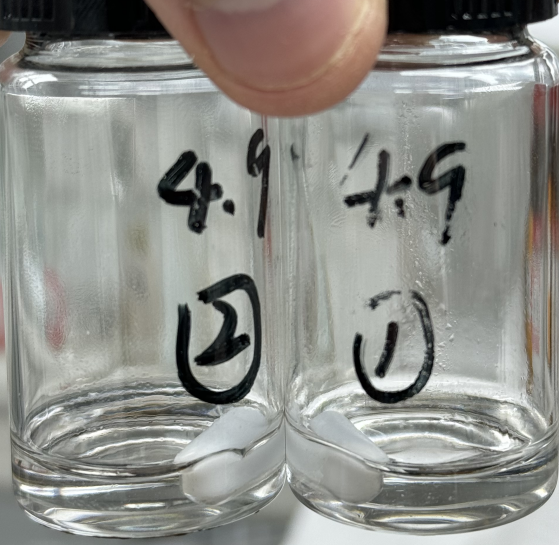

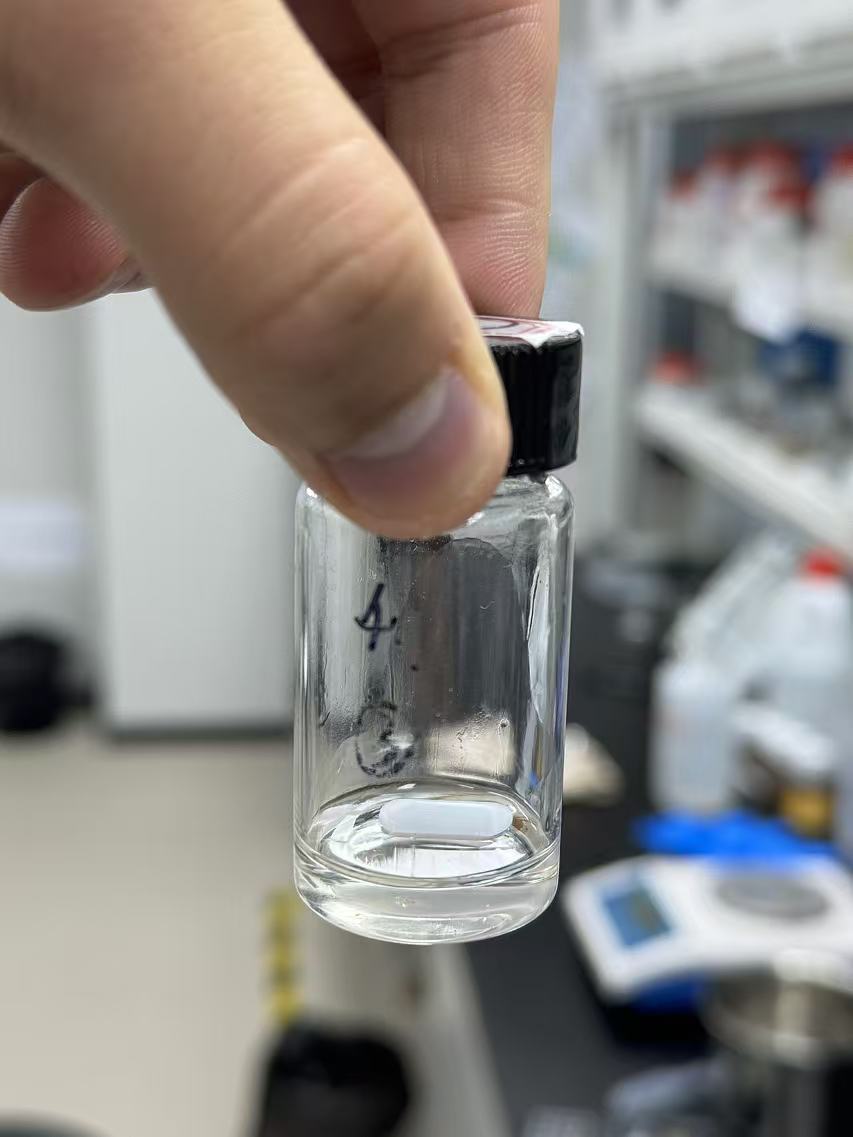


LiCl- GLY

LiCl-C_2_H_6_O_2_

**Supplementary Fig. S5** Experimental results of DES synthesis attempts using different combinations of hydrogen bond donors and acceptors


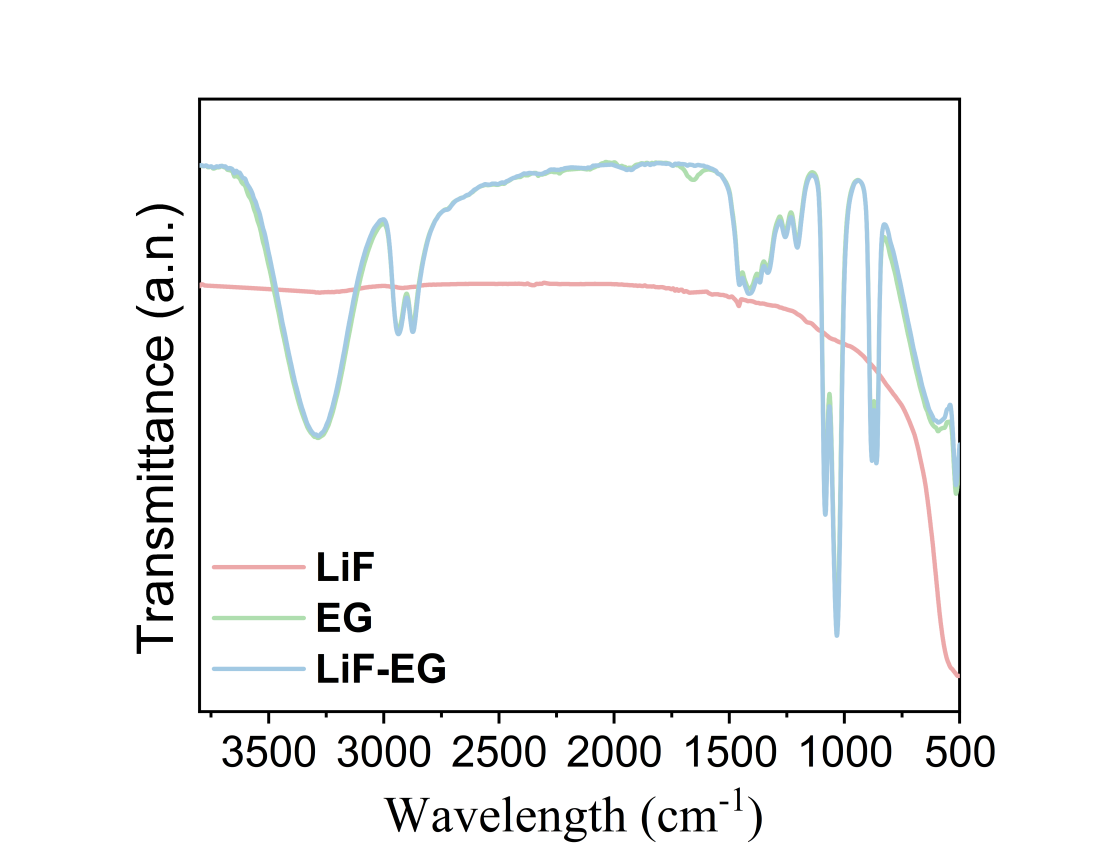


**d**

**c**

**b**

**a**


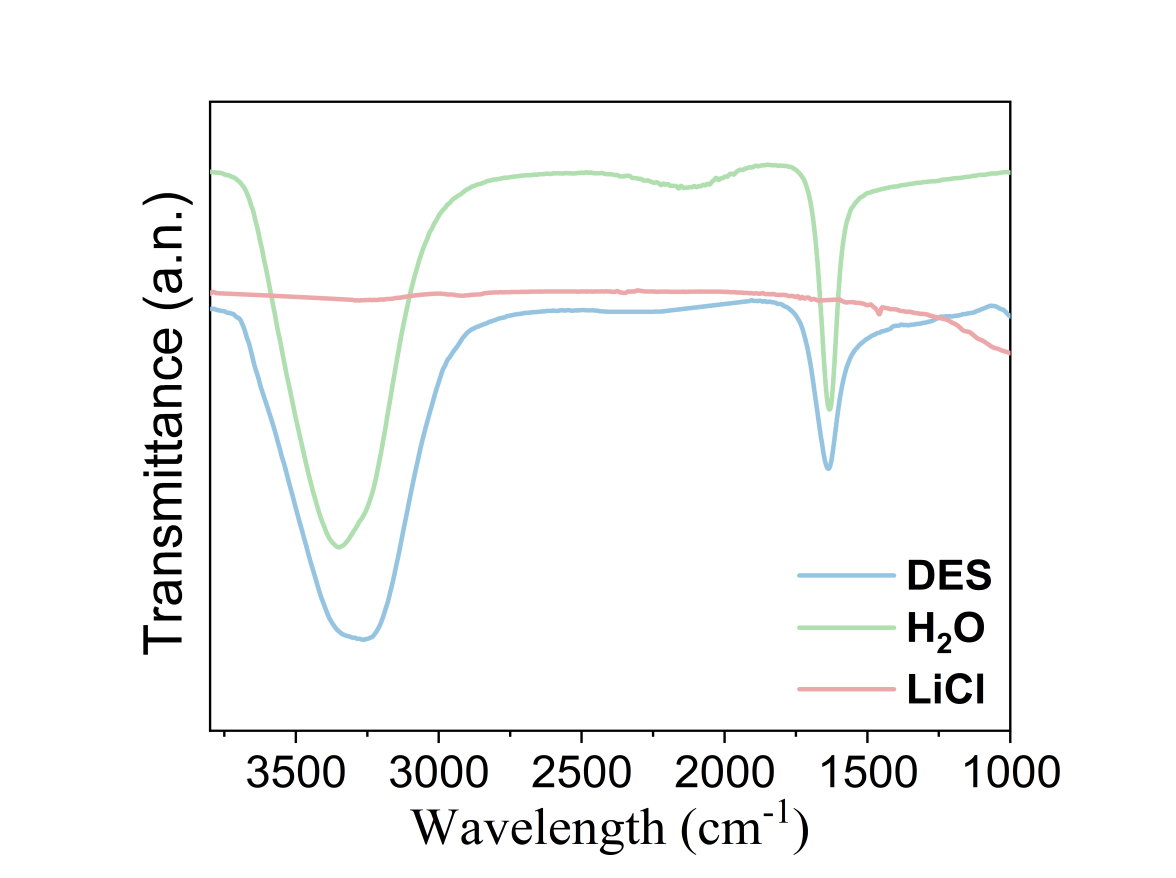

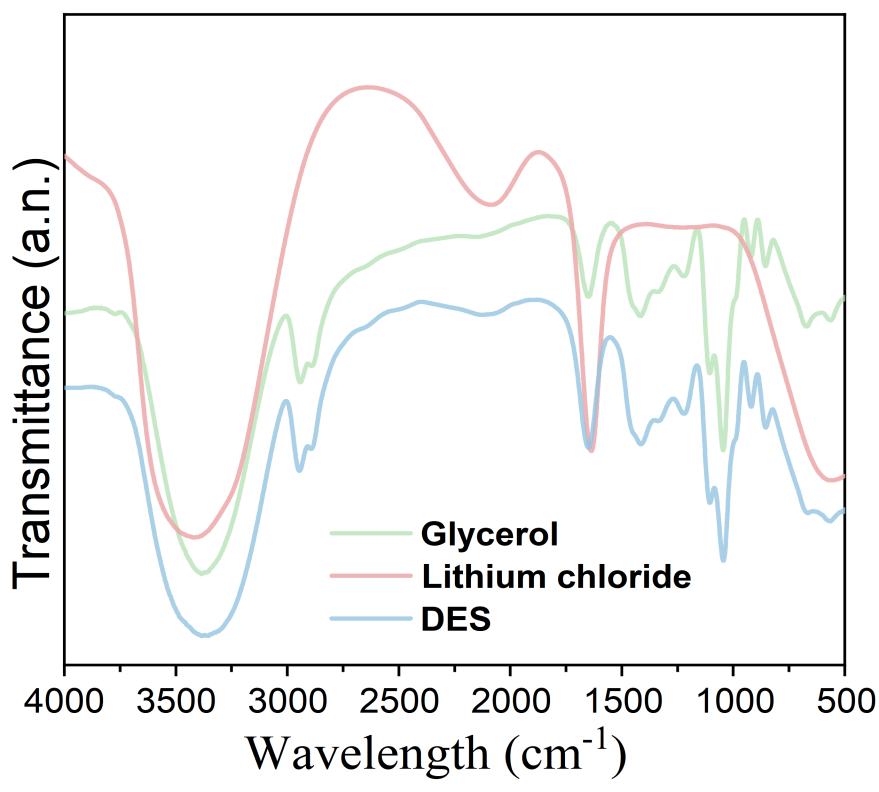


**Supplementary Fig. S6** FT-IR Test Results. a) LiCl and EG. b) LiF and EG. c) LiCl and H_2_O. d) LiCl and GLY


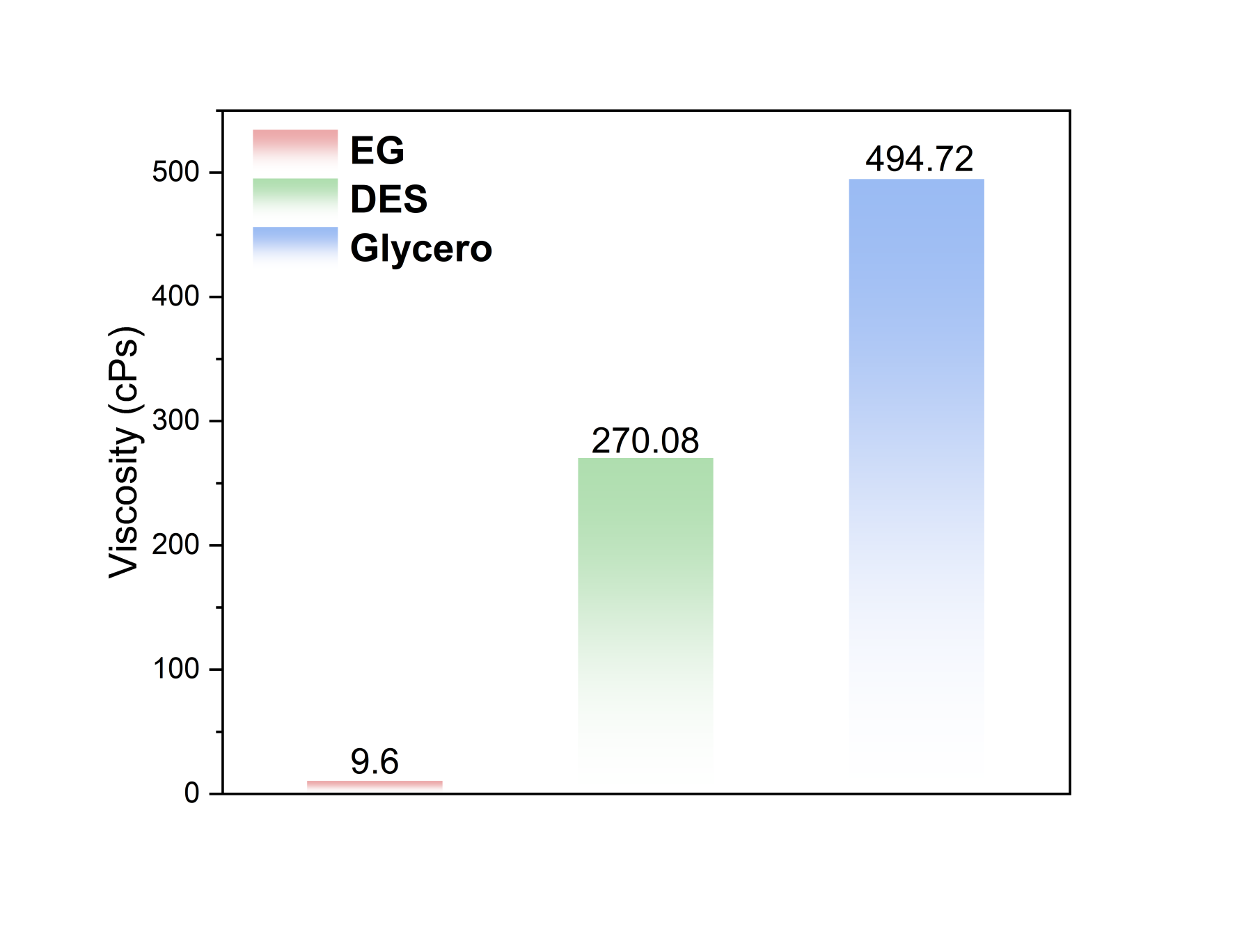


**Supplementary Fig. S7** Viscosity comparison of Ethylene Glycol, Glycerol, and DES.


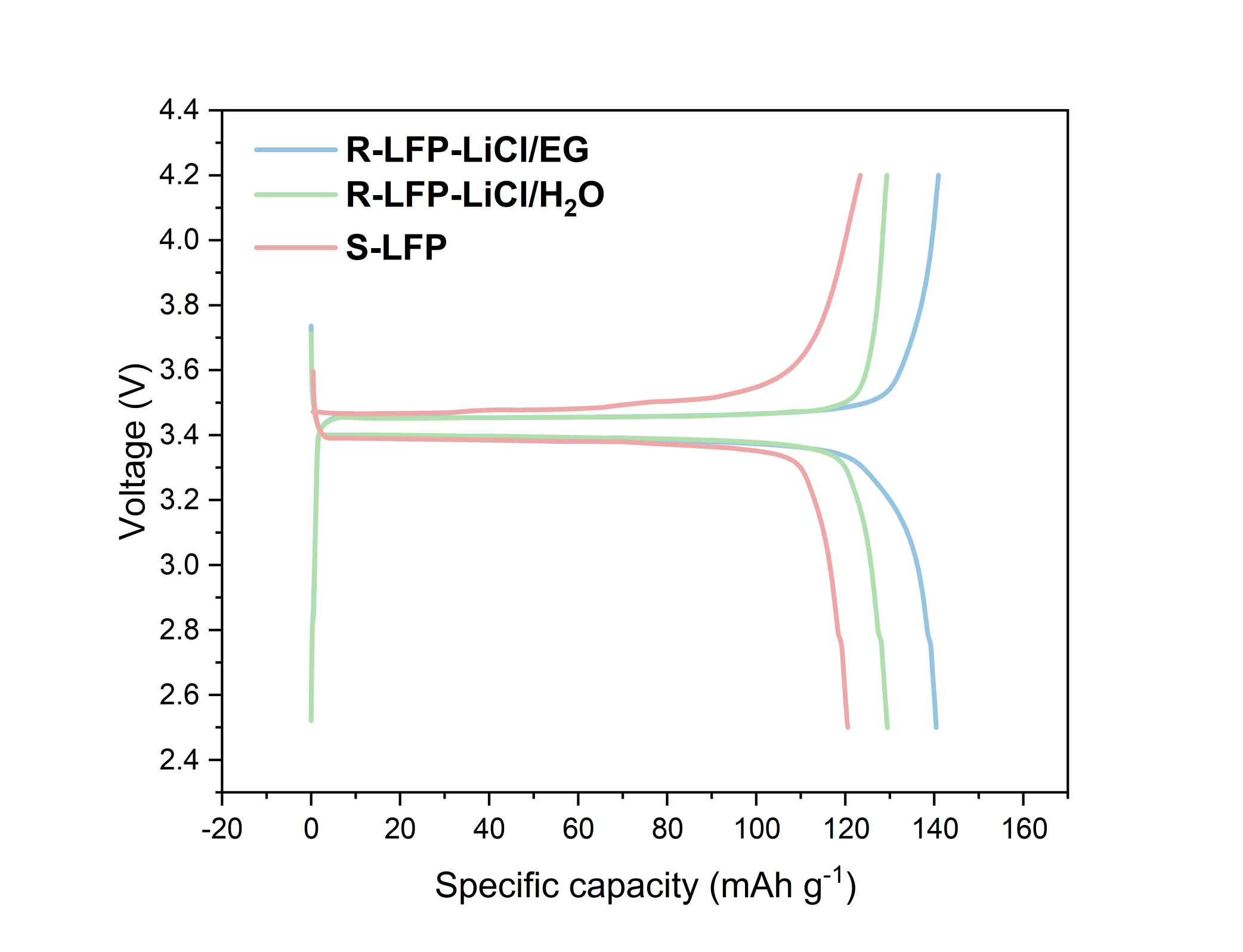


**Supplementary Fig. S8** First-cycle charge-discharge curves of R-LFP after initial restoration of S-LFP using LiCl/H₂O and LiCl/EG

**Supplementary Fig. S9** Comparison of phase transition temperatures between EG and DES via DSC.

**Supplementary Fig. S10** Thermogravimetric (TG) curve of LiCl-EG DES formation

**Supplementary Fig. S11** First-Cycle discharge specific capacity of regenerated R-LFP batteries under different processing conditions.

**Supplementary Fig. S12** TG-IR analysis results of DES.

**Supplementary Fig. S13** FT-IR Spectra of DES before and after regeneration.

**Supplementary Fig. S14** Adsorption energies of Li⁺ in different systems.

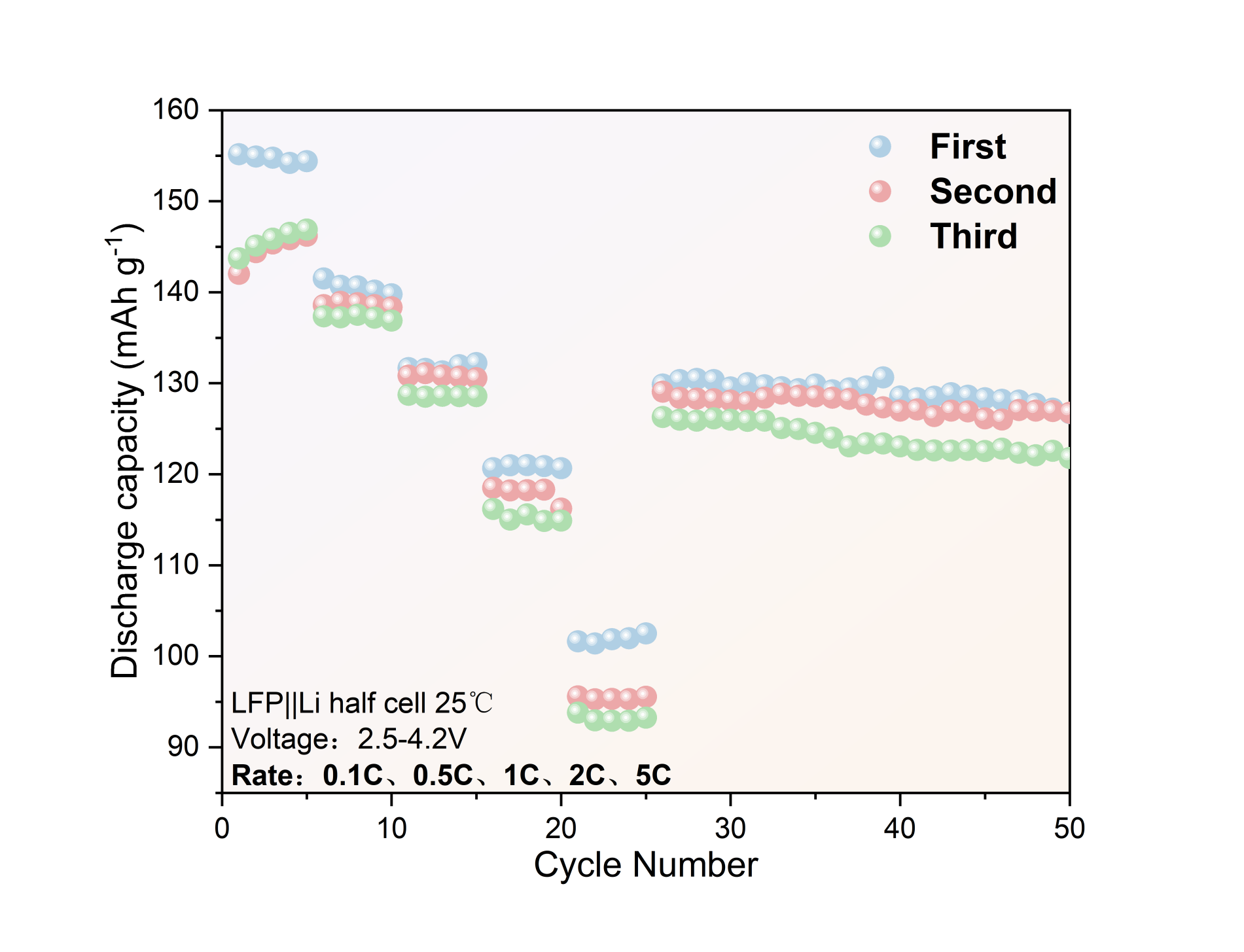


**Supplementary Fig. S15** Performance of S-LFP repaired three times with recycled DES.

**Supplementary Fig. S16** Lithium content of S-LFP after three repairs with recycled DES.


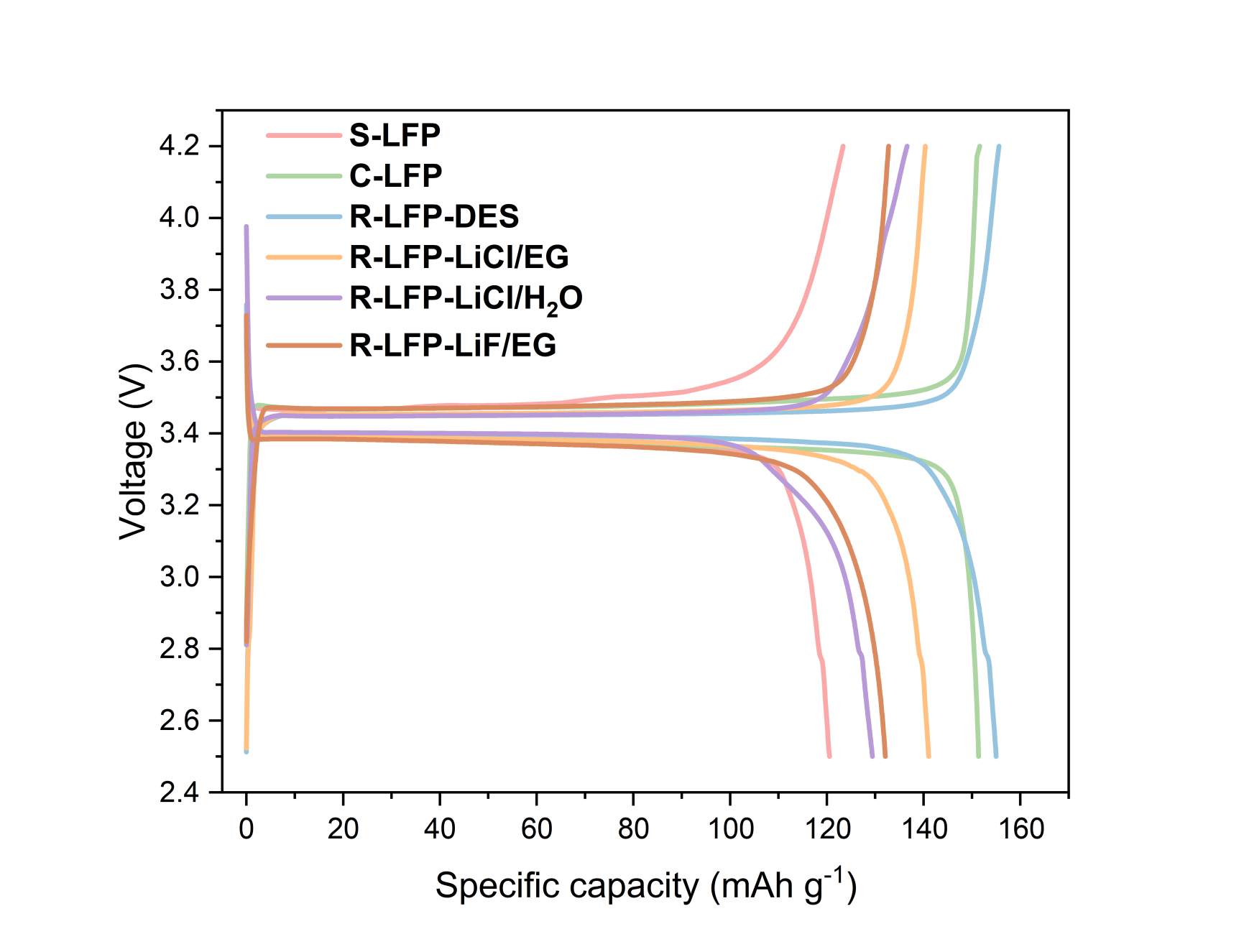

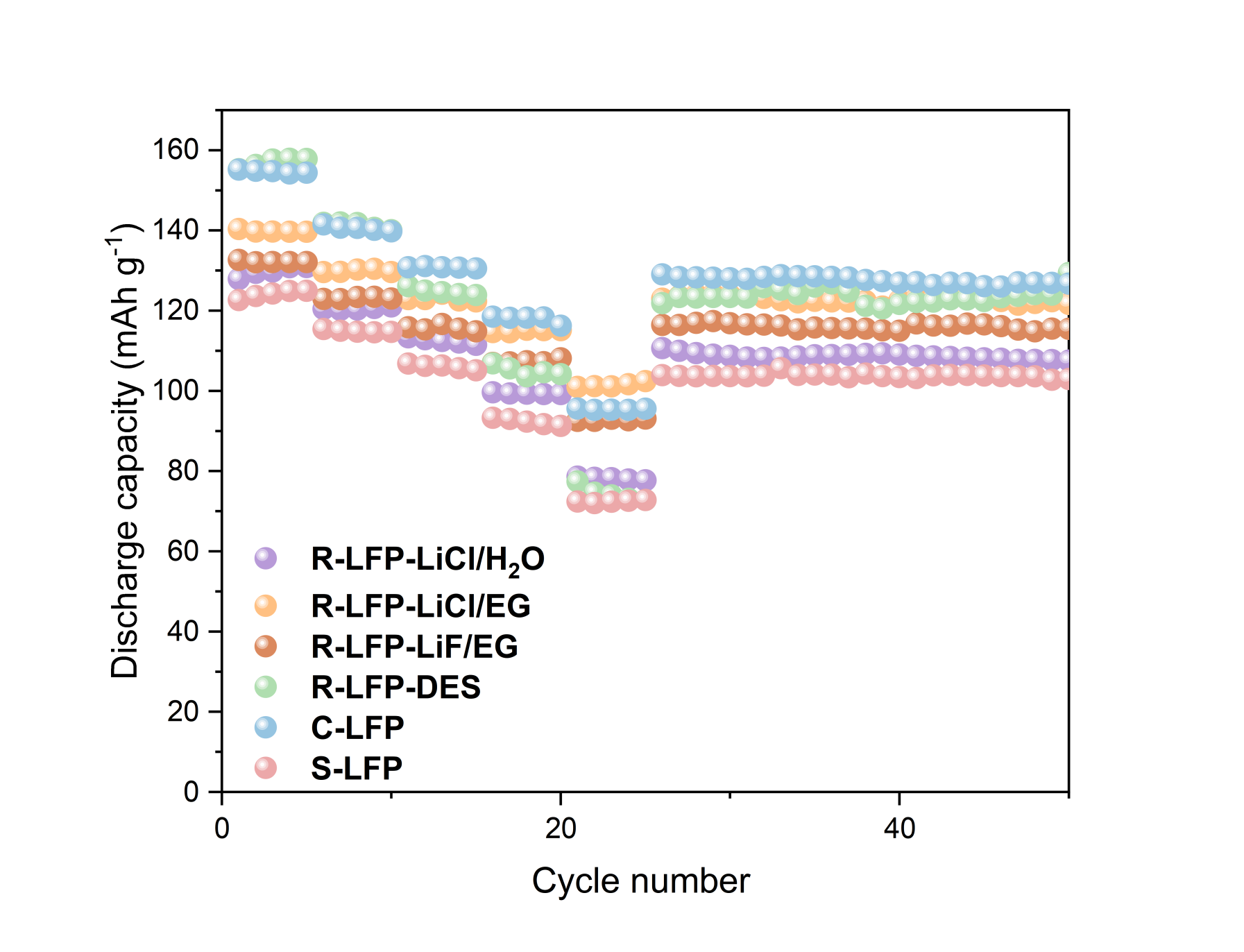


**Supplementary Fig. S17** First-cycle charge-discharge curves and rate performance of R-LFP after S-LFP restoration using different lithium salt solutions and DES systems.

**Supplementary Fig. S18** Comparison of cycling performance between S-LFP and R-LFP at 5 C high rate.

**Supplementary Fig. S19** Initial three cyclic voltammetry (CV) curves of S-LFP and R-LFP.

**Supplementary Fig. S20** CV curves of S-LFP at different scan rates.

**Supplementary Fig. S21** Comparison of diffusion coefficients between S-LFP and R-LFP using GITT.

**Supplementary Fig. S22** Initial EIS analysis of S-LFP, R-LFP, and C-LFP.

**Supplementary Fig. S23** EIS analysis of S-LFP, R-LFP, and C-LFP after 10 Charge-Discharge cycles.

**Supplementary Fig. S24** First-Cycle charge-discharge and cycling performance of S-LCO and R-LCO.

**Supplementary Fig. S25** XRD results of S-LCO and R-LCO.

**Supplementary Fig. S26** Comparison of ICP results between S-LCO and R-LCO.


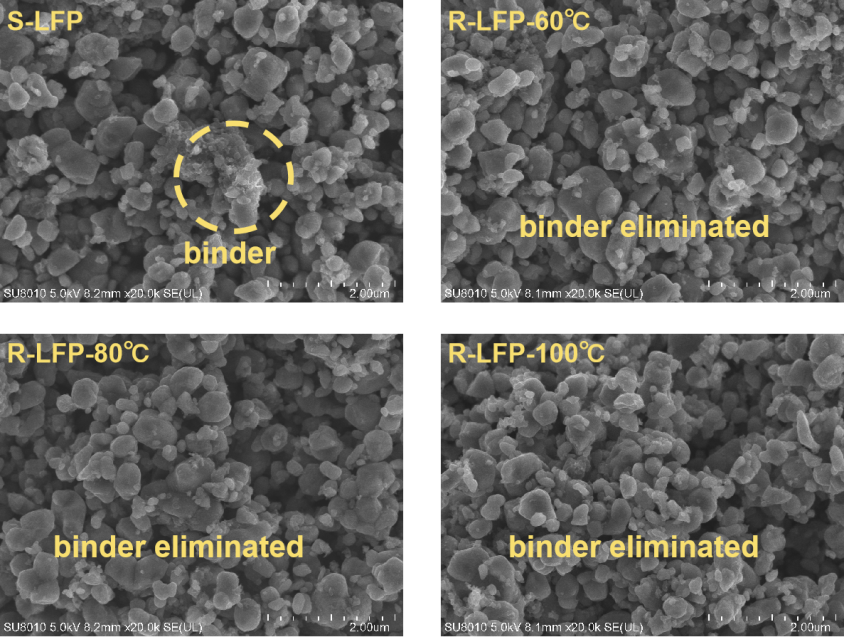


**Supplementary Fig. S27** SEM images of DES before and after regeneration.

**Supplementary Fig. S28** The conductivity of S-LFP and R-LFP

**Supplementary Fig. S29** The resistivity of S-LFP and R-LFP.

**Supplementary Fig. S30** The BET-specific surface areas of S-LFP and R-LFP


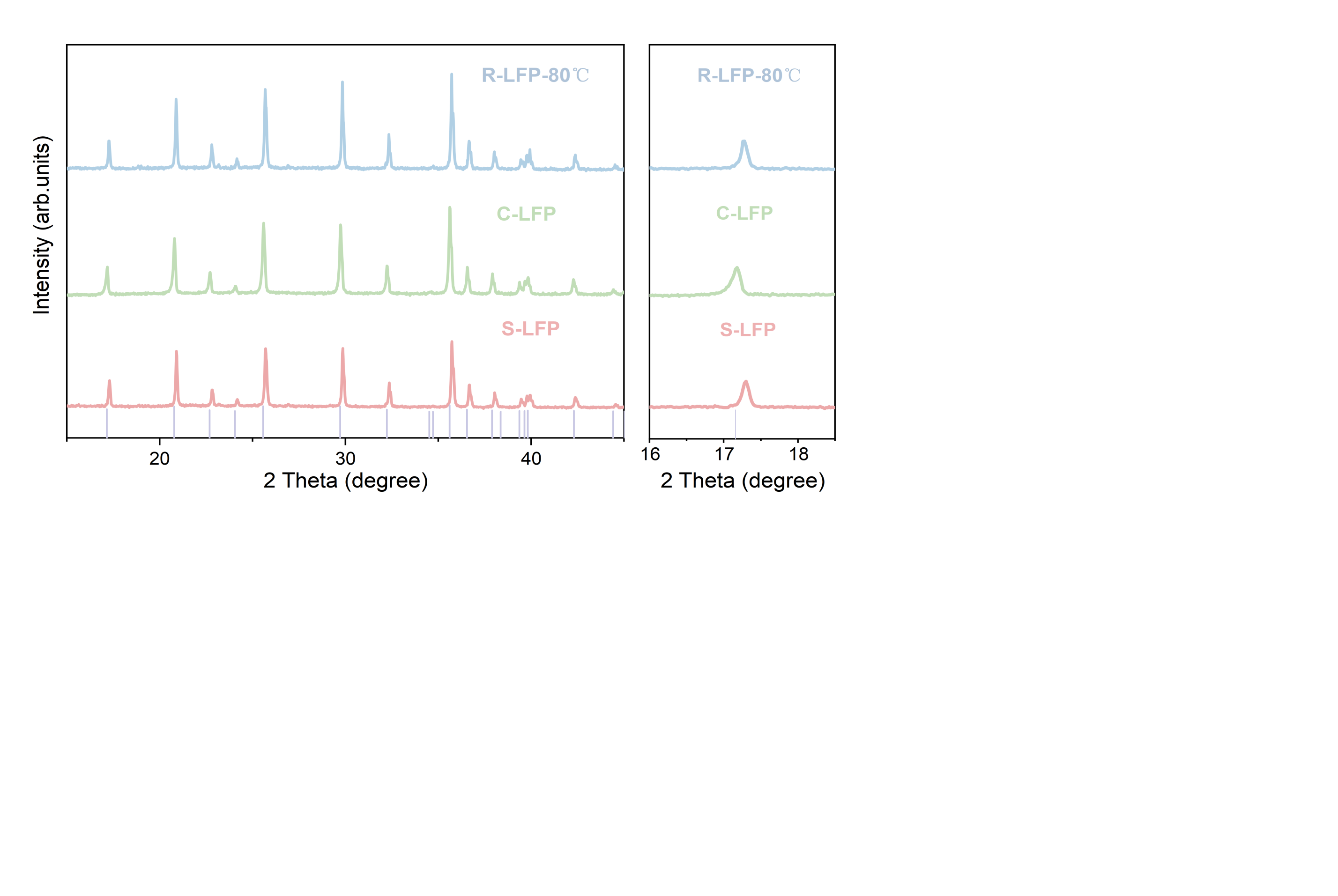


**Supplementary Fig. S31** XRD patterns of S-LFP, C-LFP, and R-LFP.

**Supplementary Fig. S32** Rietveld refinement of XRD results for R-LFP recovered at 60 ^o^C.

**Supplementary Fig. S33** Rietveld refinement of XRD results for R-LFP recovered at 100 ^o^C.

**Supplementary Fig. S34** Rietveld refinement of XRD results for C-LFP.

**Supplementary Fig. S35** Profit comparison of direct regeneration of S-LFP reported in different references^[1-5]^.


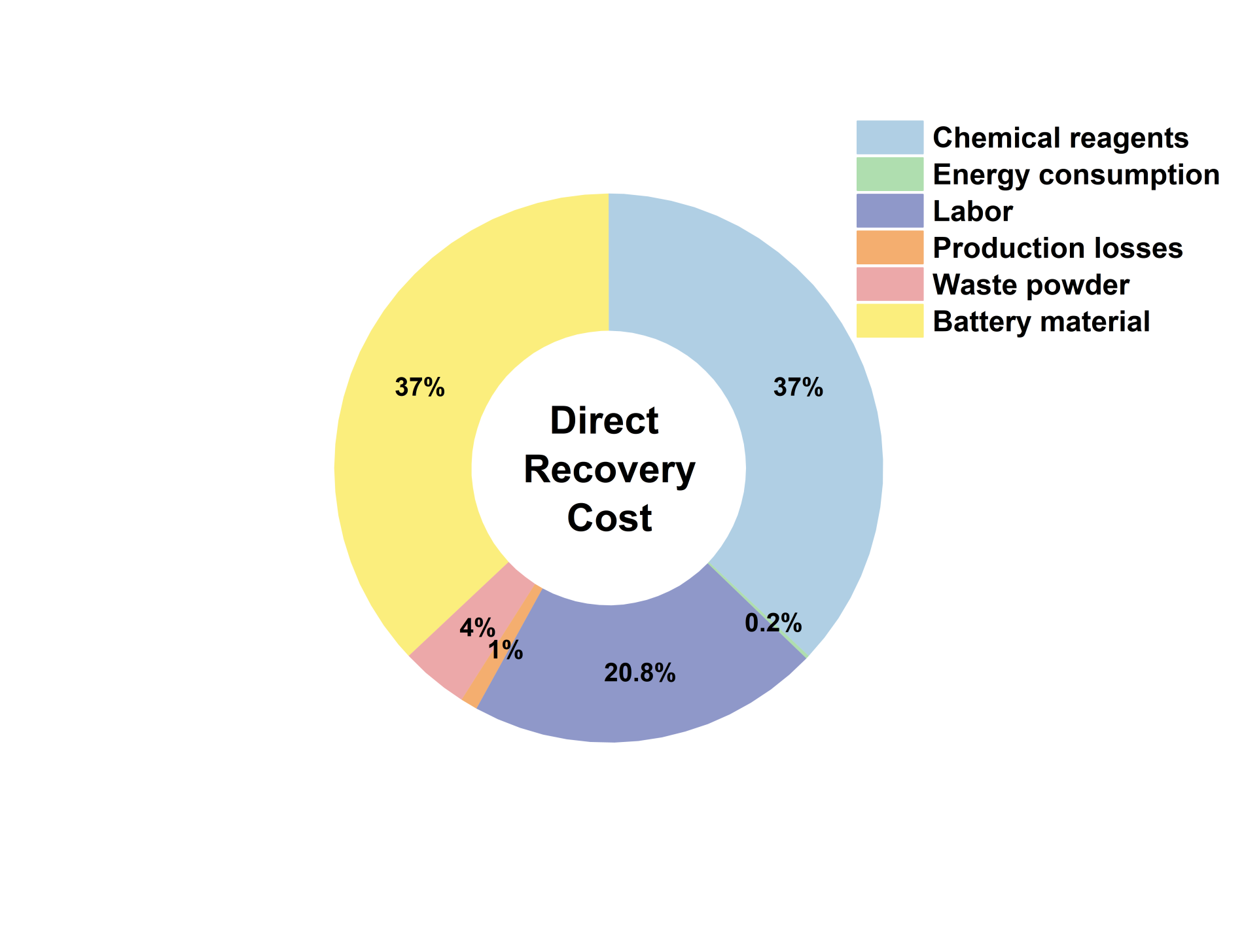


**Supplementary Fig. S36** Cost distribution of direct regeneration methods.


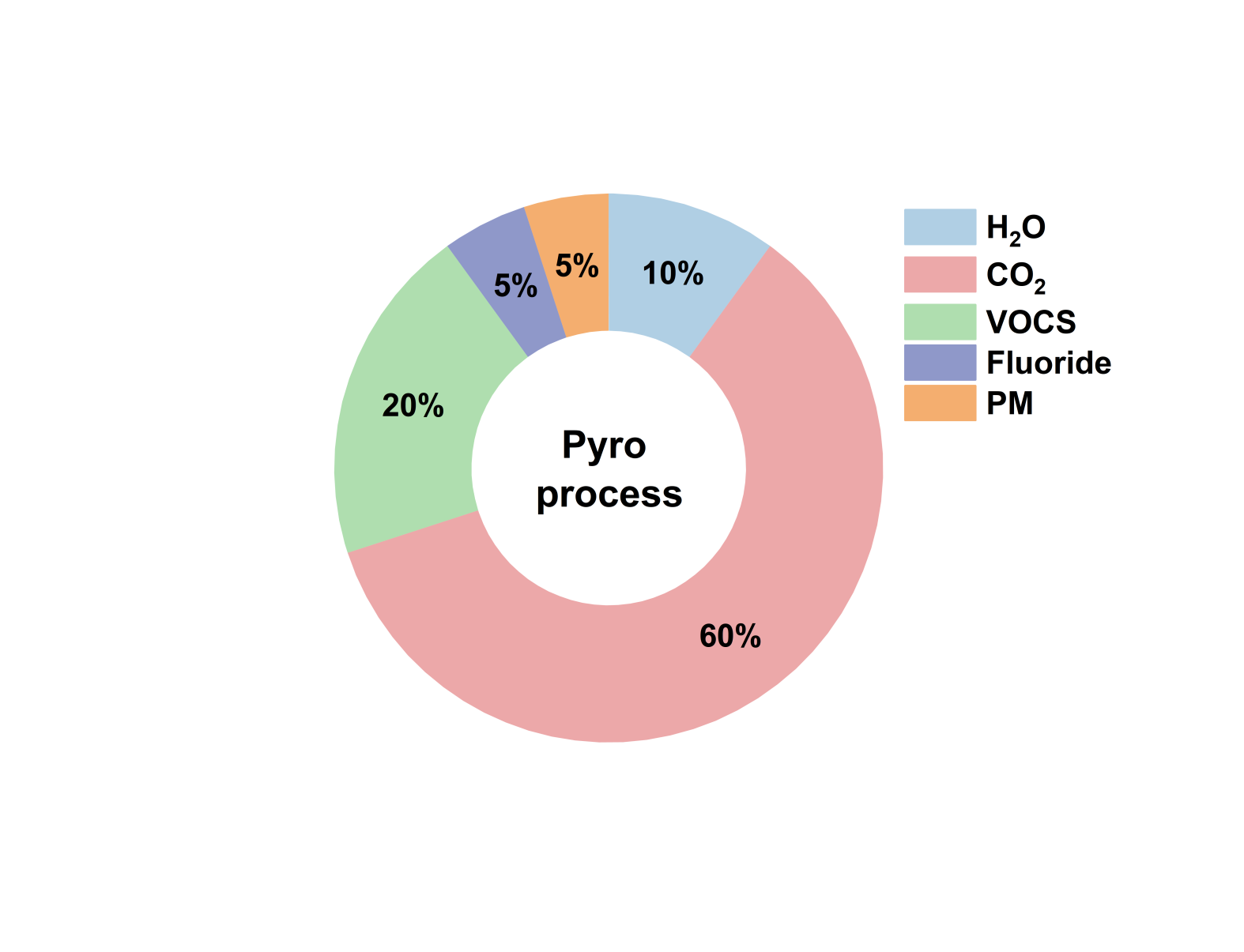


**Supplementary Fig. S37** Gas emission composition in pyrometallurgical recycling.


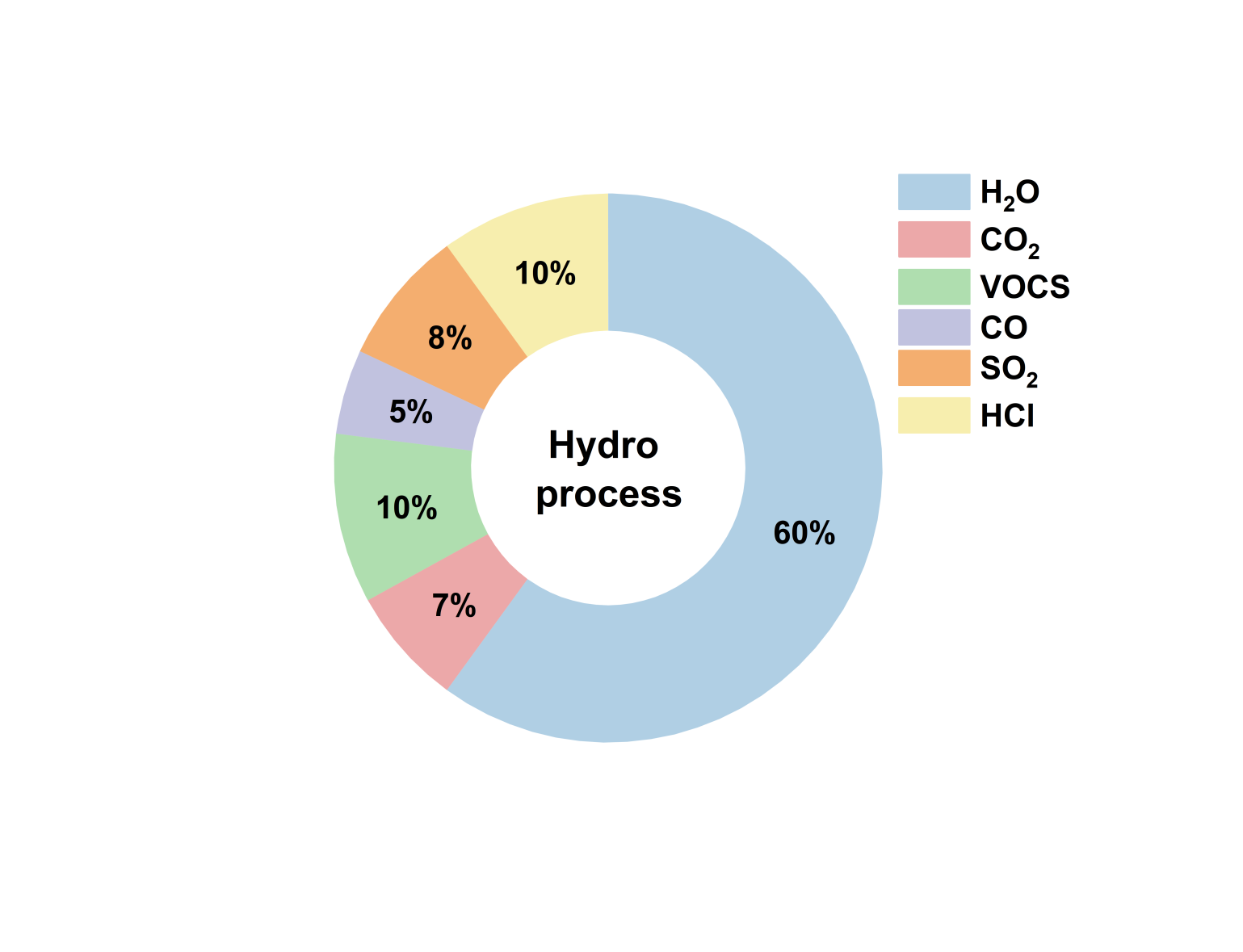


**Supplementary Fig. S38** Gas emission composition in hydrometallurgical recycling.


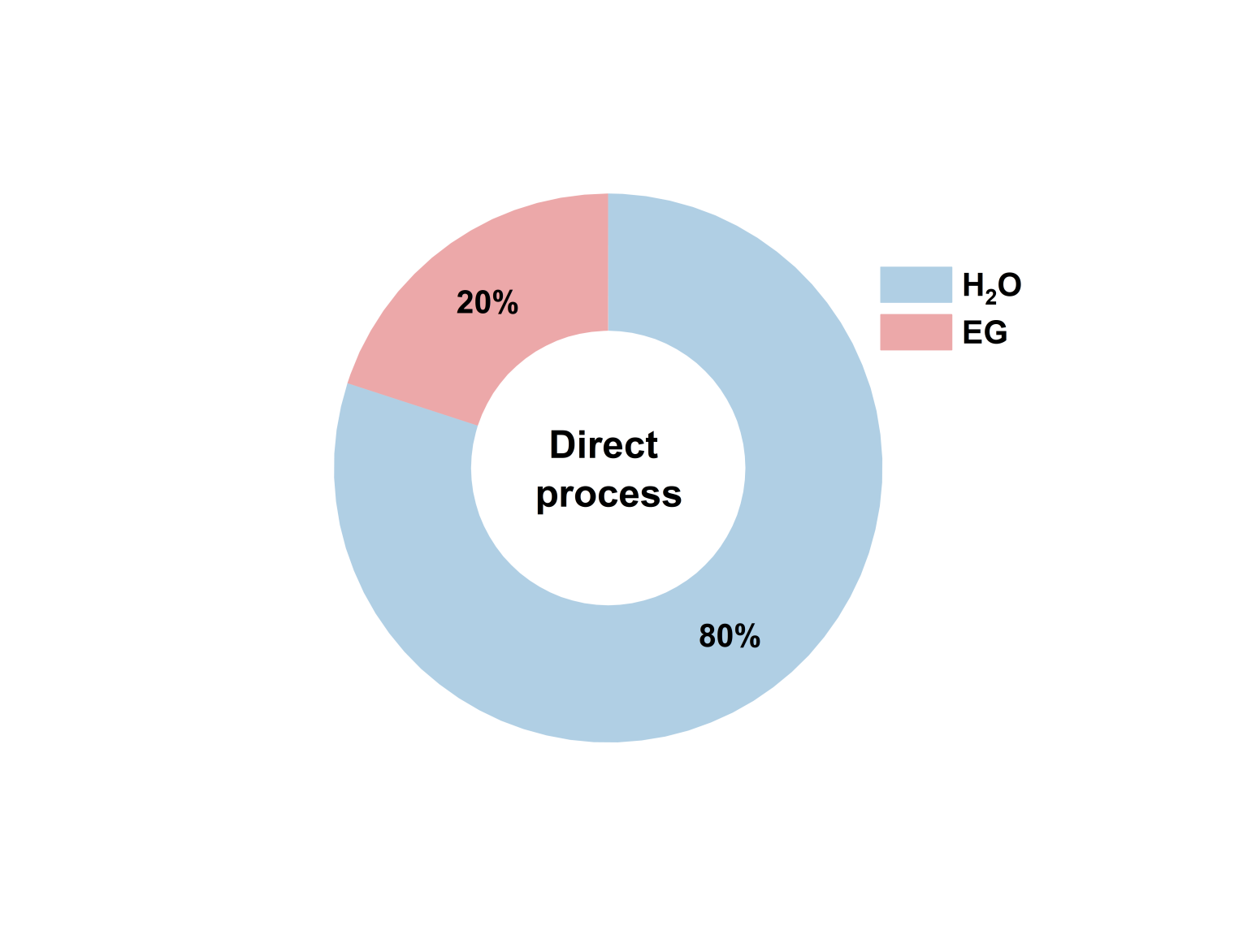


**Supplementary Fig. S39** Gas Emission Composition in Direct Regeneration Processes.


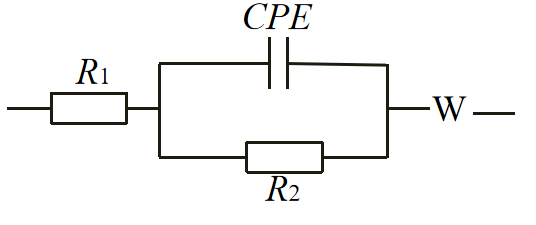


**Supplementary Fig. S40** Simulated circuit diagram for EIS fitting.

**Supplementary Table S1**. R-LFP battery performance data under different regeneration conditions.

| Molar ratio  （80 ^o^C/1：20） | | Temperature  （3：1/1：20） | | Liquid-solid ratio  （80 ^o^C/3：1） | |
| --- | --- | --- | --- | --- | --- |
| 3：1 | 155.59 mAh g^-1^ | 60 ^o^C | 141.73 mAh g^-1^ | 10：1 | 148.72 mAh g^-1^ |
| 4：1 | 144 mAh g^-1^ | 80 ^o^C | 155.59 mAh g^-1^ | 20：1 | 155.59 mAh g^-1^ |
|  | | 100 ^o^C | 134.36 mAh g^-1^ | 40：1 | 150.22 mAh g^-1^ |

**Supplementary Table S2.** Comparison of S-LFP repair data and repair temperatures reported in the literature^[1, 3, 6-18]^.

| NO. | Max Temperture (^o^C) | Residual capacity (mAh/g) | Restored capacity (mAh/g) | Upgrade rate  (%) | Cycle Rate  (C) | Cycle Number | Capacity Retention  (%) | Ref |
| --- | --- | --- | --- | --- | --- | --- | --- | --- |
| 1 | 700 | 122.5 | 151 | 23 | 1 C | 100 | 99 | [6] |
| 2 | 650 | 134.7 | 159 | 18 | 1 C | 300 | 80 | [7] |
| 3 | 300 | 134 | 162 | 20 | 1 C | 300 | 93 | [8] |
| 4 | 200 | 122 | 145 | 18 | 1 C | 100 | 99 | [9] |
| 5 | 500 | 134 | 147 | 9 | 1 C | 500 | 95 | [10] |
| 6 | 600 | 138 | 159 | 15 | 1 C | 300 | 99 | [11] |
| 7 | 180 | 138 | 155 | 12 | 1 C | 300 | 98 | [12] |
| 8 | 1100 | 140 | 153 | 9 | 1 C | 300 | 97 | [13] |
| 9 | 180 | 125 | 158 | 26 | 1 C | 500 | 84 | [14] |
| 10 | 650 | 129 | 147 | 13 | 0.5 C | 100 | 90 | [15] |
| 11 | 200 | No | 136 |  | 1 C | 300 | 98 | [16] |
| 12 | 650 | 115 | 147 | 27 | 0.1 C | 100 | 92 | [17] |
| 13 | 700 | 130. | 160. | 23 | 1 C | 200 | 94 | [18] |
| 14 | 700 | No | 145 |  | 1 C | 600 | 97 | [19] |
| 15 | 200 | No | 136 |  | 0.2 C | 100 | 100 | [20] |
| This work | 80 | 122 | 155.6 | 28 | 1 C | 300 | 93 |  |

**Supplementary Table S3.** In situ EIS fitting data for R-LFP.

| R-LFP | R_s_  (Ω) | R_ct_  (Ω) |
| --- | --- | --- |
| C-2.5 V | 1.599 | 31.59 |
| C-3.0 V | 2.01 | 31.08 |
| C-3.4 V | 1.128 | 39.39 |
| C-3.45 V | 1.67 | 38.98 |
| C-3.5 V | 1.273 | 23.08 |
| C-3.55 V | 1.682 | 17.1 |
| C-3.6 V | 1.025 | 17.09 |
| C-4.0 V | 1.366 | 13.81 |
| C-4.2 V | 1.588 | 11.493 |
| D-4.0 V | 1.952 | 11.751 |
| D-3.4 V | 1.69 | 14.25 |
| D-3.35 V | 1.325 | 33.25 |
| D-3.3 V | 1.067 | 55.54 |
| D-3.25 V | 1.3 | 62.62 |
| D-3.2 V | 1.993 | 66.25 |
| D-3.0 V | 1.175 | 65.38 |
| D-2.5 V | 1.182 | 65.88 |

**Supplementary Table S4.** In situ EIS fitting data for S-LFP.

| S-LFP | R_s_  (Ω) | R_ct_  (Ω) |
| --- | --- | --- |
| C-2.5 V | 1.789 | 75.48 |
| C-3.0 V | 1.58 | 72.87 |
| C-3.4 V | 2.32 | 71.11 |
| C-3.45 V | 2.312 | 70.36 |
| C-3.5 V | 2.32 | 56.37 |
| C-3.55 V | 2.152 | 45.36 |
| C-3.6 V | 2.36 | 47.42 |
| C-4.0 V | 2.273 | 42.03 |
| C-4.2 V | 2.368 | 35.45 |
| D-4.0 V | 2.023 | 34.26 |
| D-3.4 V | 2.164 | 32.97 |
| D-3.35 V | 2.027 | 33.71 |
| D-3.3 V | 2.239 | 56.33 |
| D-3.25 V | 2.812 | 79.31 |
| D-3.2 V | 2.033 | 84.23 |
| D-3.0 V | 1.366 | 87.9 |
| D-2.5 V | 2.948 | 112.6 |

**Supplementary Table S5.** ICP data for S-LFP, R-LFP, and C-LFP.

|  | Major elements content (wt.%) | | |  | Molar ration of | |
| --- | --- | --- | --- | --- | --- | --- |
|  | Li | Fe | P |  | Li/Fe | Li/P |
| S-LFP | 3.46 | 30.43 | 17.89 |  | 0.91 | 0.85 |
| R-LFP | 4.19 | 32.32 | 18.49 |  | 1.03 | 1.0 |
| C-LFP | 4.12 | 32.82 | 18.73 |  | 1.01 | 0.98 |

**Supplementary Table S6.** Refined XRD data for S-LFP.

| LiFePO_4_ (Space Group Pnma) | | | | | | |
| --- | --- | --- | --- | --- | --- | --- |
| Lattice  Parameters | a/Å | | b/Å | c/Å | | V/Å^3^ |
|  | 10.3198  (5) | | 6.00340  (29) | 4.69278  (24) | | 290.74  (4) |
| Li/Fe Antisite | 3.50 % | | | | | |
| Atoms | | | | | | |
| Label | Elem | | Mult | Frac | | Uiso |
| Li1 | Li^1+^ | | 4 | 0.956 | | 0.0374 |
| Fe7 | Fe^2+^ | | 4 | 0.956 | | 0.0063 |
| P1 | P | | 4 | 1.000 | | 0.0004 |
| O1 | O^2-^ | | 4 | 1.000 | | 0.0081 |
| O2 | O^2-^ | | 4 | 1.000 | | 0.0194 |
| O3 | O^2-^ | | 8 | 1.000 | | 0.0232 |
| Fe7 | Fe^2+^ | | 4 | 0.035 | | 0.0250 |
| Li8 | Li^1+^ | | 4 | 0.035 | | 0.0250 |
| Agreement factors | | | | | | |
| χ^2^ =1.49 | | R_P_=3.01 % | | | R_WP_=1.38 % | |

**Supplementary Table S7.** Refined XRD data for R-LFP-80 ^o^C.

| LiFePO_4_ (Space Group Pnma) | | | | | | |
| --- | --- | --- | --- | --- | --- | --- |
| Lattice  Parameters | a/Å | | b/Å | c/Å | | V/Å^3^ |
|  | 10.3190  (5) | | 6.00294  (30) | 4.69242 (25) | | 290.67 (4) |
| Li/Fe Antisite | 1.46 % | | | | | |
| Atoms | | | | | | |
| Label | Elem | | Mult | Frac | | Uiso |
| Li1 | Li^1+^ | | 4 | 0.986 | | 0.03956 |
| Fe7 | Fe^2+^ | | 4 | 0.986 | | 0.00485 |
| P1 | P | | 4 | 1.000 | | 0.00300 |
| O1 | O^2-^ | | 4 | 1.000 | | 0.01106 |
| O2 | O^2-^ | | 4 | 1.000 | | 0.01495 |
| O3 | O^2-^ | | 8 | 1.000 | | 0.01340 |
| Fe7 | Fe^2+^ | | 4 | 0.014 | | 0.38753 |
| Li8 | Li^1+^ | | 4 | 0.014 | | 0.02500 |
| Agreement factors | | | | | | |
| χ^2^ =1.49 | | R_P_=3.01 % | | | R_WP_=1.38 % | |

**Supplementary Table S8.** Refined XRD data for R-LFP-60 ^o^C.

| LiFePO_4_ (Space Group Pnma) | | | | | | |
| --- | --- | --- | --- | --- | --- | --- |
| Lattice  Parameters | a/Å | | b/Å | c/Å | | V/Å^3^ |
|  | 10.32050  (26) | | 6.00321  (14) | 4.69106  (14) | | 290.640  (17) |
| Li/Fe Antisite | 1.56 % | | | | | |
| Atoms | | | | | | |
| Label | Elem | | Mult | Frac | | Uiso |
| Li1 | Li^1+^ | | 4 | 0.985 | | 0.0374 |
| Fe7 | Fe^2+^ | | 4 | 0.985 | | 0.0063 |
| P1 | P | | 4 | 1.000 | | 0.0004 |
| O1 | O^2-^ | | 4 | 1.000 | | 0.0081 |
| O2 | O^2-^ | | 4 | 1.000 | | 0.0194 |
| O3 | O^2-^ | | 8 | 1.000 | | 0.0232 |
| Fe7 | Fe^2+^ | | 4 | 0.015 | | 0.0250 |
| Li8 | Li^1+^ | | 4 | 0.015 | | 0.0250 |
| Agreement factors | | | | | | |
| χ^2^ =0.95 | | R_P_=7.58 % | | | R_WP_=9.78 % | |

**Supplementary Table S9.** Refined XRD data for R-LFP-100 ^o^C.

| LiFePO_4_ (Space Group Pnma) | | | | | | |
| --- | --- | --- | --- | --- | --- | --- |
| Lattice  Parameters | a/Å | | b/Å | c/Å | | V/Å^3^ |
|  | 10.32004  (30) | | 6.00324  (16) | 4.69210  (16) | | 290.693  (18) |
| Li/Fe Antisite | 1.58 % | | | | | |
| Atoms | | | | | | |
| Label | Elem | | Mult | Frac | | Uiso |
| Li1 | Li^1+^ | | 4 | 0.985 | | 0.0374 |
| Fe7 | Fe^2+^ | | 4 | 0.985 | | 0.0063 |
| P1 | P | | 4 | 1.000 | | 0.0004 |
| O1 | O^2-^ | | 4 | 1.000 | | 0.0081 |
| O2 | O^2-^ | | 4 | 1.000 | | 0.0194 |
| O3 | O^2-^ | | 8 | 1.000 | | 0.0232 |
| Fe7 | Fe^2+^ | | 4 | 0.015 | | 0.0250 |
| Li8 | Li^1+^ | | 4 | 0.015 | | 0.0250 |
| Agreement factors | | | | | | |
| χ^2^ =1.01 | | R_P_=7.33 % | | | R_WP_=9.88 % | |

**Supplementary Table S10.** Refined XRD data for C-LFP.

| LiFePO_4_ (Space Group Pnma) | | | | | | |
| --- | --- | --- | --- | --- | --- | --- |
| Lattice  Parameters | a/Å | | b/Å | c/Å | | V/Å^3^ |
|  | 10.324043  (32) | | 6.004876  (12) | 4.692075  (16) | | 290.883  (20) |
| Li/Fe Antisite | 1.5% | | | | | |
| Atoms | | | | | | |
| Label | Elem | | Mult | Frac | | Uiso |
| Li1 | Li^1+^ | | 4 | 0.985 | | 0.0374 |
| Fe7 | Fe^2+^ | | 4 | 0.985 | | 0.0063 |
| P1 | P | | 4 | 1.000 | | 0.0004 |
| O1 | O^2-^ | | 4 | 1.000 | | 0.0081 |
| O2 | O^2-^ | | 4 | 1.000 | | 0.0194 |
| O3 | O^2-^ | | 8 | 1.000 | | 0.0232 |
| Fe7 | Fe^2+^ | | 4 | 0.015 | | 0.0250 |
| Li8 | Li^1+^ | | 4 | 0.015 | | 0.0250 |
| Agreement factors | | | | | | |
| χ^2^ =0.93 | | R_P_=9.86% | | | R_WP_=7.58% | |

**Supplementary Table S11.** Recovery cost of different LFP battery recycling (Monetary unit: $).

|  | Pyro Process | Hydro Process | Direct Process |
| --- | --- | --- | --- |
| Chemical Reagents | 14.2 | 1.5 | 127.8 |
| Energy Consumption | 28.4 | 7.1 | 1.2 |
| Labor | 14.2 | 42.6 | 71 |
| Production Losses | 7.1 | 14.2 | 3.5 |
| Waste Powder | 14.2 | 14.2 | 14.2 |
| Battery Material | 0 | 0 | 127.8 |
| Total Cost | 78.1 | 79.6 | 345.5 |

**Supplementary Table S12.** Recovery revenue of different LFP battery recycling (Monetary unit: $).

|  | Pyro Process | Hydro Process | Direct Process |
| --- | --- | --- | --- |
| Total Revenue | 85.2 | 113.6 | 525.4 |

**Supplementary Table S13.** Energy consumption of different LFP battery recycling (MJ per kg cell).

|  | Pyro Process | Hydro Process | Direct Process |
| --- | --- | --- | --- |
| Consumption | 11.06 | 9.06 | 3.56 |

**Supplementary Table S14.** Gas release of different LFP battery recycling (unit: %).

|  | Pyro Process | Hydro Process | Direct Process |
| --- | --- | --- | --- |
| EG | 0 | 0 | 20 |
| PM | 5 | 0 | 0 |
| CO | 0 | 5 | 0 |
| SO_2_ | 0 | 8 | 0 |
| HCI | 0 | 10 | 0 |
| MF_n_ | 5 | 0 | 0 |
| VOCS | 20 | 10 | 0 |
| CO_2_ | 60 | 7 | 0 |
| H_2_O | 10 | 60 | 80 |
| Total | 100 | 100 | 100 |

**Supplementary Table S15.** Adsorption energies of Li⁺ in different systems.

|  | Electrostatic  kJ mol^-1^ | Lennard-Jones  kJ mol^-1^ | Intermolecular Energy  kJ mol^-1^ |
| --- | --- | --- | --- |
| Li^+^ - Li_0.75_FePO_4_ | -22248.98 ± 5.82 | 2611.29 ± 4.25 | -19637.69 ± 1.71 |
| EG - Li_0.75_FePO_4_ | -10757.97 ± 18.27 | 316.65 ± 3.64 | -10441.32 ± 18.04 |
| Cl^-^ - Li_0.75_FePO_4_ | 18.78 ± 1.01 | -7.45 ± 0.23 | 11.33 ± 1.20 |
| Li^+^ - Cl^-^ | -57904.73 ± 50.51 | 4276.78 ± 21.00 | -53627.94 ± 35.29 |
| Li^+^ - EG | -36975.2 ± 84.03 | 4223.15 ± 14.32 | -32752.04 ± 78.79 |

**Supplementary Table S16.** Diffusion coefficients of free Li^+^ and adsorbed Li^+^.

|  | D / 10^-5^ cm^2^/s^2^ |
| --- | --- |
| Li^+^ (adsorbed) | 4.032 *10^-6^ ± 1.068*10^-6^ |
| Li^+^ (free) | 2.948*10^-5^ ± 3.174*10^-6^ |

**References**

[1] Y. Song, B. Xie, S. Song, S. Lei, W. Sun, R. Xu, Y. Yang, *Green Chemistry* **2021**, 23, 3963.

[2] K. Jia, G. Yang, Y. He, Z. Cao, J. Gao, H. Zhao, Z. Piao, J. Wang, A. M. Abdelkader, Z. Liang, R. V. Kumar, G. Zhou, S. Ding, K. Xi, *Advanced Materials* **2024**, 36.

[3] Y. Han, Y. Fang, M. Yan, H. Qiu, Y. Han, Y. Chen, L. Lin, J. Qian, T. Mei, X. Wang, *Green Chemistry* **2024**, 26, 9791.

[4] X. Qiu, C. Wang, Y. Chen, Z. Du, L. Xie, Q. Han, L. Zhu, X. Cao, X. Ji, *Small* **2024**.

[5] D. Yang, Z. Fang, Y. Ji, Y. Yang, J. Hou, Z. Zhang, W. Du, X. Qi, Z. Zhu, R. Zhang, P. Hu, L. Qie, Y. Huang, *Angewandte Chemie International Edition* **2024**, 63.

[6] X. Li, M. Wang, Q. Zhou, M. Ge, M. Zhang, W. Liu, Z. Shi, H. Yue, H. Zhang, Y. Yin, S.-T. Yang, *ACS Materials Letters* **2024**, 6, 640.

[7] Z. Wang, H. Xu, Z. Liu, M. Jin, L. Deng, S. Li, Y. Huang, *Journal of Materials Chemistry A* **2023**, 11, 9057.

[8] Y. Yang, Z. Liu, J. Zhang, Y. Chen, C. Wang, *Journal of Alloys and Compounds* **2023**, 947.

[9] D. Peng, X. Wang, S. Wang, B. Zhang, X. Lu, W. Hu, J. Zou, P. Li, Y. Wen, J. Zhang, *Green Chemistry* **2022**, 24, 4544.

[10] Z. Wang, D. Wu, X. Wang, Y. Huang, X. Wu, *Industrial & Engineering Chemistry Research* **2023**, 62, 1181.

[11] D. Tang, G. Ji, J. Wang, Z. Liang, W. Chen, H. Ji, J. Ma, S. Liu, Z. Zhuang, G. Zhou, *Adv Mater* **2024**, 36, e2309722.

[12] Y. Guo, Y. Yao, C. Guo, Y. Song, P. Huang, X. Liao, K. He, H. Zhang, H. Liu, R. Hu, W. Wang, C. Li, S. Wang, A. Nie, Y. Yuan, Y. Huang, *Energy & Environmental Science* **2024**, 17, 7749.

[13] L. Yang, B. Zhang, S. Chen, Q. Pan, W. Li, C. Gan, W. Deng, G. Zou, H. Hou, L. Yang, X. Ji, *Chem Commun (Camb)* **2024**, 60, 9384.

[14] X. Liu, M. Wang, L. Deng, Y.-J. Cheng, J. Gao, Y. Xia, *Industrial & Engineering Chemistry Research* **2022**, 61, 3831.

[15] C. Qi, S. Wang, X. Zhu, T. Zhang, Y. Gou, Z. Xie, Y. Jin, Y. Wang, L. Song, M. Zhang, *Journal of Alloys and Compounds* **2022**, 924.

[16] F. Larouche, K. Amouzegar, A. Vijh, G. P. Demopoulos, *Journal of Power Sources* **2024**, 624.

[17] G. Hu, Y. Gong, Z. Peng, K. Du, M. Huang, J. Wu, D. Guan, J. Zeng, B. Zhang, Y. Cao, *ACS Sustainable Chemistry & Engineering* **2022**, 10, 11606.

[18] Q. Sun, X. Li, H. Zhang, D. Song, X. Shi, J. Song, C. Li, L. Zhang, *Journal of Alloys and Compounds* **2020**, 818.
